# Integrated metagenomic–metabolomic insights into plant–microbe interactions mediated by *Bacillus* volatile compounds

**DOI:** 10.1128/aem.02523-25

**Published:** 2026-03-30

**Authors:** Haiqian Yang, Wei Liu, Jiwei Niu, Biao Geng, Pengfei Qiu, Hongshun Li, Junping Bao, Xin Pu, Yong Li, Xiaojing Jia, Yingxiang Sun, Yejun Han

**Affiliations:** 1 State Key Laboratory of Biopharmaceutical Preparation and Delivery, Institute of Process Engineering, Chinese Academy of Sciences 74526, Beijing, China; 2 University of Chinese Academy of Sciences 74519, Beijing, China; 3 National Engineering Research Center for Cultivated Land Protection, Sinochem Agriculture Linyi R&D Center, Linyi, China; 4 Syngenta Group China, Sinofert Holdings Limited 243833 , Beijing, China; The University of Arizona, Tucson, Arizona, USA

**Keywords:** *Bacillus subtilis*, volatile compounds, root microbiota, plant–microbe interaction, metagenome, metabolome

## Abstract

Modulation of plant–microbe interactions with signaling molecules offers a promising strategy to promote plant growth and stress adaptation. However, identifying effective signaling molecules and elucidating the mechanisms for regulating the rhizosphere microbiome remain major challenges. In this study, the roles and mechanisms of *Bacillus* volatile compounds as potential signaling molecules in plant–microbe interactions were investigated. First, the genome and metabolism of a novel *Bacillus subtilis* strain capable of producing acetoin and 2,3-butanediol were studied, and the titers of the two compounds were increased to 86.76 g/L by sequential metabolic engineering. Subsequently, the effects of volatile compounds on the growth of vegetables (*Brassica rapa* and *Solanum lycopersicum* var.) were studied. Plant growth, nutrient (nitrogen, phosphorus, and potassium) utilization efficiency, and salt stress resistance were improved significantly. Compared with water as a control, significant changes in the abundance of 109 microbial genera of *B. rapa*’s rhizosphere microbiome were identified with volatile compound application. Notably increased microbes included nitrogen-fixing, phosphate- and potassium-solubilizing, stress-resistant, plant growth-promoting, and auxin-secreting microbes. Additionally, genes involved in nitrogen, phosphorus, and potassium utilization in the rhizosphere microbiome were significantly increased, and corresponding metabolism was found. Finally, metabolomic analyses of *S. lycopersicum* var.’s roots and leaves revealed 67 significantly upregulated compounds with the application of volatile compounds. These compounds were primarily involved in stress resistance, oxidative stress alleviation, free radical scavenging, and auxin-related plant growth promotion. This work demonstrates that *Bacillus* volatile compounds regulate rhizosphere microbiome and plant–microbe interactions and enhance plant nutrient utilization efficiency, stress tolerance, and growth.

**IMPORTANCE:**

Plant productivity and stress resilience are strongly influenced by interactions between plants and the rhizosphere microbiome, yet practical strategies to rationally modulate native soil microbial communities remain limited. This study demonstrates that *Bacillus* volatile compounds, specifically acetoin and 2,3-butanediol, function as effective signaling molecules that coordinate plant–microbe interactions in the rhizosphere. By integrating plant physiology, metagenomics, and metabolomics, we show that these volatile compounds not only enhance plant growth and nutrient use efficiency but also reprogram rhizosphere microbial communities toward functions that benefit nitrogen, phosphorus, and potassium acquisition and stress adaptation. Notably, volatile application improved plant salt tolerance, highlighting their strong ecological and physiological impact. This work provides mechanistic evidence that *Bacillus*-derived volatiles act as signaling molecules to activate the rhizosphere microbiome and plant metabolic responses. The findings offer a scalable and environmentally friendly strategy for improving crop performance and soil health, with broad implications for sustainable agriculture.

## INTRODUCTION

The plant rhizosphere microbiome constitutes a complex and dynamic community that plays a pivotal role in regulating plant growth, nutrient acquisition, and resilience to environmental stresses. These microorganisms modulate plant physiology through multiple plant–microbe interaction mechanisms, including nitrogen fixation, phosphate solubilization, siderophore production, and phytohormone biosynthesis. Moreover, certain rhizosphere microorganisms enhance plant tolerance to biotic and abiotic stresses by inducing systemic resistance or regulating antioxidant pathways (1).

The rhizosphere microbiome holds great promise for promoting plant growth, improving stress tolerance, and reducing dependence on chemical fertilizers (2). Although these microorganisms naturally inhabit most agricultural soils, their ecological functions are often constrained by environmental conditions. A wide range of agricultural and environmental factors—such as intensive chemical fertilizer application, pesticide overuse, and soil salinization—can disrupt rhizosphere microbial structure and function, thereby impairing beneficial plant–microbe interactions. While chemical fertilizers are indispensable for maintaining global food production, the excessive application of nitrogen (N), phosphorus (P), and potassium (K) fertilizers has resulted in low nutrient use efficiency and various agroecological problems. High N inputs lead to nitrogen loss and water eutrophication, P fertilizers are readily fixed in soil and become unavailable, and imbalanced K fertilization reduces crop stress resilience and overall quality (3). Pesticide overuse further disturbs microbial community composition, suppresses beneficial taxa, and diminishes key microbial functions required for plant health. Additionally, soil salinity—exacerbated by poor irrigation management and climate-induced aridification—damages soil structure, decreases microbial diversity, and limits root colonization by beneficial microorganisms (4). Collectively, these disturbances weaken plant–microbe interactions, ultimately constraining plant growth, nutrient uptake, and resilience (5). Thus, developing sustainable strategies that protect or restore the rhizosphere microbiome is essential for improving crop productivity under modern agricultural challenges.

The application of exogenous microbial inoculants has demonstrated benefits in enhancing nutrient acquisition, improving stress tolerance, inhibiting pathogens, and increasing plant salt resistance. As a result, microbial inoculants have attracted growing interest as promising alternatives to reduce fertilizer and pesticide inputs. However, their performance in agricultural soils remains inconsistent. In complex soil environments, introduced microorganisms often exhibit low survival rates and are frequently outcompeted by native microbial communities, resulting in limited or variable effectiveness (6, 7).

In natural systems, plant root exudates shape rhizosphere microbial composition and activity by supplying carbon sources, signaling molecules, and selective pressures that favor beneficial taxa. Rhizosphere microorganisms, in turn, produce metabolites—including volatile compounds—that participate in plant–microbe communication and defense regulation. Targeted modulation of the rhizosphere microbiome through signaling molecules represents a promising approach for activating native beneficial microbes and improving plant–microbe compatibility. Volatile compounds are particularly attractive as potential signaling molecules for modulating rhizosphere microbiome structure and promoting plant growth. Although many rhizosphere microbes synthesize volatile compounds, their natural production is often limited due to environmental and climatic constraints (8). The regulatory effects of volatile compounds on the rhizosphere microbiome and plant–microbe interactions, as well as their potential applications in crop cultivation, remain largely unexplored. With advances in metagenomics and other multi-omics technologies, deeper insights can now be gained into how volatile compounds interact with the rhizosphere microbiome and plant metabolic pathways. Understanding these interactions may enable the development of microbiome-modulating strategies using volatile compounds to support sustainable agriculture, including biofertilizers, biostimulants, and microbial consortia designed to enhance crop productivity and resilience.

This study was initiated to investigate the effects of two volatile compounds—acetoin and 2,3-butanediol—produced by a metabolically engineered *Bacillus subtilis* strain, on vegetable physiology and the underlying mechanisms from both rhizosphere microbiome and plant metabolism perspectives. First, a mutant strain derived from wild-type *B. subtilis* was engineered to enhance volatile compound production. Second, its effects on plant growth, nutrient uptake, and salt stress resistance were evaluated. Subsequently, the rhizosphere microbiome and its associated metabolic pathways and gene expression profiles were analyzed. In addition, plant metabolomic profiles were examined to elucidate the interactions among volatile compounds, rhizosphere microbes, and the host plant. Overall, this study provides new insights into plant–microbe interactions mediated by *Bacillus*-derived volatile compounds through integrated metagenomic and metabolomic analyses, providing a theoretical basis for their application in agricultural production.

## MATERIALS AND METHODS

### Reagents, strains, and instruments

The reagents used in this study are listed as follows: yeast extract (Oxoid), hydrogen peroxide, potassium chloride, acetoin (Aladdin), ethanol, 2,3,5-triphenyltetrazolium chloride, tryptone, sodium chloride, ammonium sulfate, dipotassium phosphate, magnesium chloride, manganese chloride, glucose, sodium hydroxide, hydrochloric acid, 2,3-butanediol (Aladdin), sodium bicarbonate, ammonium molybdate, ascorbic acid, sulfuric acid, ammonium acetate, potassium dichromate, ferrous sulfate, o-phenanthroline, urea (46% N), monocalcium phosphate (46% P_2_O_5_), potassium sulfate (50% K_2_O), methanol, acetonitrile, formic acid, Liquid Chromatography-Mass Spectrometry (LC-MS) grade water, and LC-MS-grade isopropanol (Fisher Scientific). All chemicals used were purchased from Sinopharm Chemical Reagent Co., Ltd. (SCRC) with analytical grade unless otherwise specified.

*Brassica rapa* var. and *Solanum lycopersicum* var. were used as plant materials. The strain *Bacillus subtilis* AC-6 was obtained from our laboratory collection. The instruments applied in this study include UHPLC-Q Exactive high-resolution mass spectrometer (Thermo Fisher Scientific), BEH C18 column (Waters, USA, 100 mm × 2.1 mm i.d., 1.7 µm), BEH Amide column (Waters, USA, 100 mm × 2.1 mm i.d., 1.7 µm), JXDC-20 nitrogen blow concentrator (Shanghai Jingxin Industrial Development Co., Ltd.), LNG-T88 desktop centrifugal concentrator (Huamei Biochemical Instrument Factory, Taicang), Wonbio-96c high-throughput tissue grinder (Shanghai Wanbo Biotechnology Co., Ltd.), SBL-10DT ultrasonic cleaner 300W–10L (Ningbo Xinzhi Biotechnology Co., Ltd.), Centrifuge 5430R (Eppendorf, Germany), NewClassic MF MS105DU electronic balance (Mettler Toledo, Switzerland).

### Whole-genome sequencing of *B. subtilis* AC-6

*B. subtilis* AC-6 was cultured in Luria-Bertani (LB) medium at 37°C and 200 rpm until logarithmic phase. Cells were collected by centrifugation at 10,000 rpm at 4°C. After liquid nitrogen grinding, genomic DNA was extracted via lysis in SDS (sodium dodecyl sulfate) buffer containing proteinase K and β-mercaptoethanol, chloroform/isoamyl alcohol extraction, isopropanol precipitation, and 75% ethanol washing, followed by purification and quality assessment (9). Libraries were prepared using SQK-LSK110 (Oxford Nanopore Technologies) and VAHTS Universal Plus DNA Library Prep Kit for MGI V2/Illumina V2 (Vazyme, China), followed by sequencing on the Nanopore PromethION and Illumina NovaSeq 6000 platforms. Raw data were assessed with GUPPY (v5.0.16) and assembled using Unicycler (v0.5.0).

### Construction of *B. subtilis* engineering strains and plasmids

The strains and plasmids applied for the metabolic engineering of *B. subtilis* are listed in Table S1. *Escherichia coli* Trans1-T1 and recombinant *B. subtilis* strains were cultivated in LB medium at 37°C with shaking at 200 rpm. Ampicillin, kanamycin, and erythromycin were used for plasmid and transformant selection at final concentrations of 50, 25, and 10 μg/mL, respectively. *B. subtilis* AC-6 (Bac)-competent cells were first prepared and transformed with plasmid pMK4-comK via electroporation (2 KV, 5 ms) (10). Subsequently, the competence regulator gene *comK* was induced, enabling homologous recombination (11, 12) to knock out the *acoA* (acetoin dehydrogenase subunit alpha) and *eutD* (phosphate acetyltransferase) genes from the *B. subtilis* AC-6 genome (Bac/pMK4-comK, Bac01). Using *B. subtilis* AC-6 genomic deoxyribonucleic acid (DNA) as the template, upstream and downstream homologous arms (approximately 1,000 bp) of the *acoA* and *eutD* genes were polymerase chain reaction (PCR)-amplified. The antibiotic resistance gene (*ermC*) was PCR-amplified from plasmid pMD19T-aea-T7P. The homologous arms and the resistance cassette were fused using overlap PCR to generate linear DNA fragments for *acoA*/*eutD* gene replacement. The linear DNA fragments were transformed into *B. subtilis*-competent cells, and the resulting transformants were identified by genomic PCR amplification and sequencing.

The expression plasmid pMA5-HpaII-alsS-HpaII-alsD-bdhA was constructed as follows: the *alsS* gene (acetolactate synthase), *alsD* gene (α-acetolactate decarboxylase), and *bdhA* gene ((R, R)-butanediol dehydrogenase) were PCR-amplified using *B. subtilis* AC-6 genomic DNA as the template. The *alsD* and *bdhA* genes were fused via overlap PCR to obtain the *alsD-bdhA* gene cluster. These DNA fragments were assembled into the expression vector pMA5-HpaII using Gibson assembly (TransGen Biotech, China) to generate pMA5-HpaII-alsS-HpaII-alsD-bdhA. The recombinant plasmid was verified by PCR using primers pMA5-test-F (5′-AATATAAAGTATAGTGTGTTAT-3′) and pMA5-test-R (5′-GCTGCATGTGTCAGAGGTTTTCA-3′).

### Fermentation of *Bacillus subtilis* for acetoin and 2,3-butanediol production

The *B. subtilis* AC-6 and its engineered strains were inoculated into LB medium and cultured at 37°C, 200 rpm for 16 h to obtain the first-stage seed culture, a 4% (vol/vol) inoculum was then transferred to 25 mL of LB medium and cultured at 37°C, 200 rpm. The shake-flask fermentation medium (25 mL in 250 mL flasks) contained 120 g/L glucose, 7.5 g/L yeast extract, 5.0 g/L (NH_4_)_2_SO_4_, 1 g/L K_2_HPO_4_, 0.75 g/L MgCl_2_, and 0.075 g/L MnCl_2_, with an initial pH of 5.5.

Fed-batch fermentation of the engineered *B. subtilis* strains was conducted in 2.5 L working-volume fermenters (5 L total volume). The seed culture was prepared by cultivating the engineered strains in LB medium for 12 h, followed by reinoculation into fresh LB medium at 10% (vol/vol) inoculation for an additional 8 h. The seed culture was then transferred to the fermentation medium at 10% (vol/vol) inoculation. Fermentation was carried out at 37°C, 600 rpm, and 20% dissolved oxygen, with pH maintained at 5.5 using 5 M NaOH and 30% H_3_PO_4_. A glucose stock solution (500 g/L) was sterilized at 115°C for 15 min, added to the fermentation medium to reach an initial glucose concentration of 60 g/L, and fed intermittently during fermentation. The fermentation medium contained: 15 g/L peptone, 7.5 g/L yeast extract, 10 g/L NaCl, 7.5 g/L (NH_4_)_2_SO_4_, 1.5 g/L K_2_HPO_4_, 0.75 g/L MgCl_2_, and 0.05 g/L MnCl_2_, sterilized at 115°C for 15 min. The fermentation was terminated when the glucose was nearly depleted. The culture was then heated at 60°C for 30 min to inactivate the bacteria, after which the supernatant was collected by centrifugation and stored at 4°C.

### Application of acetoin and 2,3-butanediol mixture in *B. rapa* cultivation

Brown soil from Linyi, Shandong (34.95°N, 118.46°E) was used in the pot experiment, with the following characteristics: pH 8.16, alkaline nitrogen 73.7 mg/kg, available phosphorus 16.5 mg/kg, available potassium 92 mg/kg, and organic matter 9.68 g/kg. The soil was sieved (5 mm), homogenized, and loaded into each pot with 4.0 kg. Each pot was watered with 500 mL, and five *B. rapa* seeds were sown evenly (3 cm apart), then covered with 0.5 cm of soil. *B. rapa* was grown in a glasshouse at 25°C under light-emitting diode (LED) light (25 W) from 7:00 am to 19:00. The fermentation broth was heated at 60°C to inactivate the *Bacillus*, and the supernatant was collected by centrifugation for the activity assay. Ten days after emergence, two uniform seedlings were retained per pot. Each pot was irrigated with 500 mL of *Bacillus* fermentation mixture (diluted 300 times: F×300, 1,000 times: F×1,000, 3,000 times: F×3,000), and water was used as the control. Each treatment had six replicates. A second application of the fermentation mixture was performed on day 20, and destructive sampling was conducted on day 30 to weigh the aboveground fresh biomass of *B. rapa*. In the preliminary experiments, we compared water and LB medium (diluted 1,000 times and 3,000 times) as additives for their effects on the growth of *B. rapa*. No differences were observed between the two treatments; therefore, water was used as the control in subsequent experiments.

### Nutrient utilization analysis in *B. rapa* cultivation

The cultivation of *B. rapa* was the same as described above, with five treatments and six replicates each: (i) N_0_PK (with no nitrogen); (ii) NP_0_K (with no phosphorus); (iii) NPK_0_ (with no potassium); (iv) NPK (full fertilization); and (v) NPK + F × 1000 (full fertilization + fermentation mixture of acetoin and 2,3-butanediol diluted 1,000 times on day 10 and 20) (13). The fertilizer application was based on 60 kg/acre of 15-15-15 compound fertilizer and 150,000 kg/acre of topsoil, with 4.0 kg of soil per pot, corresponding to 0.24 g of each nutrient (N, P_2_O_5_, K_2_O) per pot. The fertilizers applied included urea, calcium dihydrogen phosphate, and potassium sulfate, which were ground and mixed with soil. After 30 days, *B. rapa* was harvested, inactivated at 105°C for 30 min, dried at 75°C until constant weight, ground (<2 mm), and analyzed for total N, P, and K. The samples were first treated with H_2_SO_4_–H_2_O_2_, followed by nitrogen (13), phosphorus (14), and potassium (15) analysis.

Nutrient use efficiency (16) was calculated as follows:

Nitrogen use efficiency (NUE) = [(DW × N%) − (N_0_PK DW × N%)]/N applied × 100%

Phosphorus use efficiency (PUE) = [(DW × P%) − (NP_0_K DW × P%)]/P applied × 100%

Potassium use efficiency (KUE) = [(DW × K%) − (NPK_0_ DW × K%)]/K applied × 100%

### Salt stress assay in *B. rapa* cultivation

Nutritional soil (Brown Sphagnum peat, KEKKILA, Finland) was applied for *B. rapa* cultivation and salt stress assays. The soil had a particle size of 0–6 mm, pH 5.9, total nitrogen content (as N) of 0.90 mg/kg, total phosphorus (as P_2_O_5_) of 0.72 mg/kg, total potassium (as K_2_O) of 1.74 mg/kg, moisture content of 55%, and a dry bulk density of 95 g/L. Before use, the soil was autoclaved at 121°C for 20 min, cooled, and then thoroughly mixed with sterile water in a 1:2 (wt/wt) ratio. The mixture was evenly packed into pots (90 mm × 90 mm × 100 mm, length × width × height). Three to five *B. rapa* seeds were sown per pot and covered with a 0.5 cm layer of the same nutritional soil. *B. rapa* was grown in a greenhouse at 25°C with supplemental LED lighting (25 W) from 7:00 AM to 19:00. On day 5 post-emergence, one uniform seedling per pot was retained and irrigated with equal volumes of fermentation mixture (F×300, F×1,000, or F×3,000). The control group was irrigated with sterile water. On day 10 post-emergence, *B. rapa* was subjected to salt stress by irrigating each pot with 10% NaCl solution, resulting in a final soil NaCl concentration of 7.5 mg/g dry substrate. On day 15, the leaf damage index was recorded. Leaf injury was categorized as follows: Grade 0, no visible damage; Grade 1, obvious leaf curling and wilting, but leaves remain green; Grade 2, leaf curling accompanied by yellowing and wilting (17).

The damage index was calculated as follows: leaf damage index = ∑(number of leaves per grade × grade value)/(total number of investigated leaves × 2)× 100% (18).

On day 30, destructive sampling was conducted to measure the fresh weight of the above-ground biomass of *B. rapa*. The content of chlorophyll and carotenoid (19) and relative conductivity (20) of *B. rapa* were then measured. Each treatment included 18 biological replicates and was randomly divided into three pooled groups for testing.

### Metagenomic analysis of rhizosphere soil in *B. rapa* cultivation

Rhizosphere soil was collected at day 30 from three randomly selected *B. rapa* per treatment. Samples were stored at −80°C. Total DNA was extracted using the EZNA Soil DNA Kit (Omega Bio-tek), quantified with a NanoDrop 2000 (Thermo Fisher Scientific, Inc.), and checked on a 1% agarose gel. Metagenomic sequencing was performed on the Illumina HiSeq 4000 platform after fragmentation (350 bp) and PE library construction. Quality control was conducted with Fastp (v0.20.0), assembly with MEGAHIT (v1.1.2), ORF prediction with MetaGene, and annotation using Diamond (v0.8.35) against the NR and KEGG databases. Data were analyzed on the Majorbio Cloud Platform (21).

### Application of acetoin and 2,3-butanediol mixture in *S. lycopersicum* var. cultivation

Nutritional soil (as described above) was autoclaved at 121°C for 20 min and mixed with sterile water at a 1:2 (wt/wt) ratio. The mixture was packed into seedling trays with 4 × 8 wells, and one *S*. *lycopersicum* var. seed was sown per well. Trays were covered with film to reduce water evaporation and incubated in a 25°C greenhouse for seedling preparation. On day 15 post-emergence, uniform seedlings were transplanted into pots filled with 4.0 kg of soil. *S. lycopersicum* var. was grown in a 25°C greenhouse with 25 W LED lighting from 7:00 AM to 19:00. On the day of transplantation, 500 mL of fermentation mixture (F×300, F×1,000, and F×3,000) was applied. The control group received 500 mL of sterile water. Each treatment and control had six replicates. A second application of the fermentation mixture was performed on day 25 post-emergence using the same method. On day 35 post-emergence, destructive sampling was carried out. Fresh weight of the above-ground parts of *S. lycopersicum* var. was recorded.

### Untargeted metabolomics of *S. lycopersicum* var.

The pot experiment was the same as described above. On days 28, 30, and 33 post-emergence, leaf tissue was collected from the third lateral branch near the apex. Leaf surface was rinsed with purified water to remove debris, cut into ~1 cm² pieces, wrapped in foil, flash-frozen in liquid nitrogen for 5 min, and stored at −80°C. Root tips (0.5–1.0 cm) were similarly sampled, rinsed, dried, weighed, frozen, and stored. Six biological replicates per treatment were prepared. Samples were sent to Shanghai Majorbio Bio-pharm Technology Co., Ltd. for analysis. LC-MS/MS Analysis: Metabolite profiling was conducted using UHPLC-Q Exactive Orbitrap MS (Thermo Fisher Scientific). Chromatographic separation was performed on a BEH C18 column (100 mm × 2.1 mm, 1.7 µm) with mobile phase A (2% acetonitrile with 0.1% formic acid) and mobile phase B (acetonitrile with 0.1% formic acid) at a flow rate of 0.40 mL/min and 40°C column temperature. Mass spectrometry was operated in both positive and negative ionization modes, scanning in the 70–1,050 m/z range. Raw LC-MS data were processed with Progenesis QI (Waters Corporation) and matched to the MJDBPM plant metabolite database. Differential metabolites were annotated and mapped to metabolic pathways using the KEGG database (https://www.kegg.jp/kegg/pathway.html) (22).

### Analytical and statistical methods

Available nitrogen was measured using the alkaline diffusion method. Available phosphorus was extracted with sodium bicarbonate and measured via the molybdenum blue method. Available potassium was extracted with ammonium acetate and measured using flame photometry. Organic matter was determined via chemical oxidation following K_2_Cr_2_O_7_-H_2_SO_4_ digestion. Soil pH was measured at a 1:2.5 soil-to-water ratio (23). One-way ANOVA followed by Tukey’s HSD test (*P* < 0.05) was performed to assess differences in plant phenotypic traits. Data were expressed as means ± standard deviation (SD), with at least three biological replicates per experiment. GraphPad Prism 9 was used for data visualization. Glucose, acetoin, and 2,3-butanediol concentrations were quantified using high-performance liquid chromatography (HPLC) equipped with a refractive index detector (LC-20AT, Shimadzu, Japan) and Hi-Plex H column (300 mm × 7.7 mm, Agilent Technologies). The column was maintained at 65°C, with 5 mM H_2_SO_4_ as the mobile phase at a flow rate of 0.6 mL/min.

## RESULTS

### Whole-genome analysis of *Bacillus subtilis* AC-6 capable of producing acetoin and 2,3-butanediol

**Fig 1:**
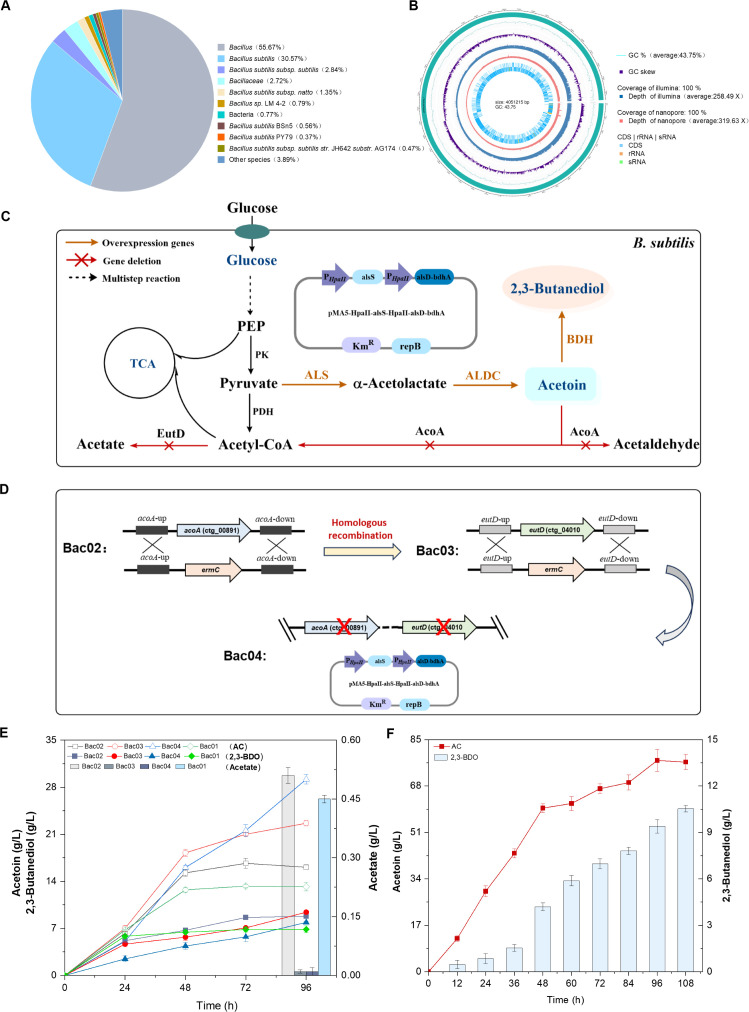
Genome annotation and metabolic engineering of *Bacillus subtilis* AC-6. (**A**) The species identification of NR (Non-Redundant) annotation. (**B**) Whole-genome circle map of *B. subtilis* AC-6. (**C**) Metabolic engineering of *B. subtilis* AC-6 to convert glucose to acetoin (AC) and 2,3-butanediol (2,3-BDO). PK, pyruvate kinase; PDH, pyruvate dehydrogenase; EutD, phosphate acetyltransferase; AcoA, acetoin dehydrogenase subunit alpha; ALS, acetolactate synthase; ALDC, α-acetolactate decarboxylase; BDH, (*R*, *R*)-butanediol dehydrogenase. (**D**) Schematic diagram of metabolic engineering. Bac02 was an engineered strain by deleting the *acoA* gene in Bac01. Bac03 was an engineered strain by deleting the *eutD* gene from the engineered strain Bac02. Bac04 was obtained by transforming the engineered strain Bac03 with the plasmid pMA5HpaIIalsSHpaIIalsDbdhA. (**E**) Production of acetoin, 2,3-butanediol, and acetate in shake flask fermentation of different engineered strains. (**F**) Production of acetoin, 2,3-butanediol in a 5 L bioreactor with strain Bac04.

In our previous research (24), *B. subtilis* AC-6, which is resistant to high temperatures (52°C) and is capable of synthesizing acetoin and 2,3-butanediol, was obtained by isolation and screening from soil, followed by combined mutagenesis. In the present study, the genome of the strain was sequenced, and the sequencing results are shown in Table S2. The raw genome sequence data have been deposited in the Genome Sequence Archive (GSA: CRA031468) at the National Genomics Data Center, China National Center for Bioinformation. Annotation in the NR database confirmed that the strain is *B. subtilis* (Fig. 1A), which is consistent with the 16S rRNA alignment results. The genome size of *B. subtilis* AC-6 is 4,051,215 bp, with a GC content of 43.75%. The genome contains 4,301 coding genes, with a total length of 3,636,105 bp, including 10 rRNA genes and 87 tRNA genes. The complete genomic map of *B. subtilis* AC-6 is shown in Fig. 1B; the features such as GC content, coding sequences (CDS), and non-coding RNA regions (rRNA, tRNA) are included. The predicted gene sequences were compared using the KEGG database BLAST+ (Version: 2.11.0+), and the functional genes were annotated (Fig. 1A). A total of 1,293 genes were annotated in the KEGG database, classified into five major categories (23 subcategories). The largest proportion of annotated genes was in the metabolism category (746 genes), of which 262 genes are involved in carbohydrate metabolism, indicating that *B. subtilis* AC-6 has strong carbohydrate metabolic capabilities. The COG database shows that 337 genes are related to carbohydrate metabolism and transport (Fig. S1B).

Based on the gene function annotations in databases such as KEGG (Kyoto Encyclopedia of Genes and Genomes) and COG, and combining the metabolic pathways in the model strain *B. subtilis* 168 (25), the key genes involved in acetoin and 2,3-butanediol metabolism in *B. subtilis* AC-6 were analyzed and annotated, including the *alsS* gene (COG0028), *alsD* gene (COG3527), and *bdhA* gene (COG0022). Pyruvate is a key intermediate for acetoin synthesis, and the glycolysis pathway in *B. subtilis* AC-6 was annotated (ko00010). After glucose enters the glycolysis pathway and synthesizes the intermediate pyruvate, acetoin is synthesized under the catalysis of ALS and ALDC and further converted to 2,3-butanediol. This is consistent with the metabolic pathway in the acetoin-producing strain *B. subtilis* 168. Additionally, key enzyme genes in the acetoin degradation pathway (acetoin dehydrogenase α subunit, COG1071) and byproduct acetate synthesis pathway (phosphate acetyltransferase, COG1207) were annotated (Fig. 1C).

### Metabolic engineering of *Bacillus subtilis* AC-6 for enhanced production of acetoin and 2,3-butanediol

Through screening and mutagenesis, a *B. subtilis* AC-6 strain with improved acetoin and 2,3-butanediol production was obtained. However, certain by-products were still detected in the fermentation broth, and the titer remained suboptimal. To further increase the production of acetoin and 2,3-butanediol, a series of metabolically engineered *B. subtilis* strains was constructed based on the native metabolic pathways of *B. subtilis* AC-6. Under aerobic conditions, *B. subtilis* also catabolizes acetoin during the stationary phase via a degradation pathway, using it as a carbon and energy source during sporulation (26). Acetoin is metabolized by acetoin dehydrogenase subunit alpha, encoded by the *acoA* gene, to form acetyl-CoA and acetaldehyde (Fig. 1C). Therefore, to prevent acetoin degradation and promote its accumulation, the *acoA* gene was knocked out from *B. subtilis* AC-6, generating the engineered strain Bac02 (Fig. 1D). After 72 h of shake flask fermentation, Bac02 accumulated 16.68 g/L acetoin, representing a 25.46% increase compared to the wild-type strain Bac01 (Fig. 1E). This result indicates that the deletion of the *acoA* gene reduced acetoin degradation, thereby enhancing acetoin accumulation.

Acetate is a major by-product produced during aerobic fermentation by *B. subtilis* (27). The acetate synthesis rate is directly correlated with cell growth and glucose consumption rates. In Bac02, acetate accumulation was also detected in the early stages of fermentation (e.g., 0.80 g/L at 24 h). Redirecting carbon flux from acetate synthesis toward acetoin and 2,3-butanediol production may thus enhance target product yields. One direct approach to reduce acetate formation is to eliminate phosphate acetyltransferase, encoded by the *eutD* gene, which catalyzes the conversion of acetyl-CoA to acetate. By deleting the *eutD* gene, the engineered strain Bac03 was constructed (Fig. 1D). Shake flask fermentation results showed that Bac03 exhibited significantly reduced acetate levels (0.01 g/L), representing a 98% reduction compared to Bac02. In addition, Bac03 accumulated 22.64 g/L acetoin after 96 h of fermentation, a 40.70% increase relative to Bac02 (at 96 h), indicating that *eutD* deletion effectively minimized acetate accumulation and enhanced acetoin production.

The synthesis of acetoin and 2,3-butanediol is dependent on the expression of key rate-limiting enzymes such as ALS (acetolactate synthase), ALDC (α-acetolactate decarboxylase), and BDH ((R, R)-butanediol dehydrogenase) (25). To further channel pyruvate into the acetoin and 2,3-butanediol biosynthesis pathway, the plasmid pMA5-HpaII-alsS-HpaII-alsD-bdhA was introduced into Bac03 to generate the engineered strain Bac04 (Fig. 1D). After 96 h of shake flask fermentation, Bac04 accumulated 29.18 g/L acetoin, which was 1.29-fold and 2.21-fold higher than Bac03 and Bac01, respectively (Fig. 1E). The concentration of 2,3-butanediol reached 7.89 g/L, with minimal acetate accumulation. Fed-batch fermentation of Bac04 was further performed in a 5 L bioreactor to produce acetoin and 2,3-butanediol. By systematically adjusting fermentation parameters (e.g., dissolved oxygen, substrate concentration, pH), with rotational speed and aeration controlled to maintain 20% dissolved oxygen. As shown in Fig. 1F, after 96 h of fermentation, Bac04 achieved acetoin and 2,3-butanediol titers of 77.36 g/L and 9.40 g/L, respectively.

### Promotion of *B. rapa* and *S. lycopersicum* var. growth by acetoin and 2,3-butanediol fermentation mixture

**Fig 2:**
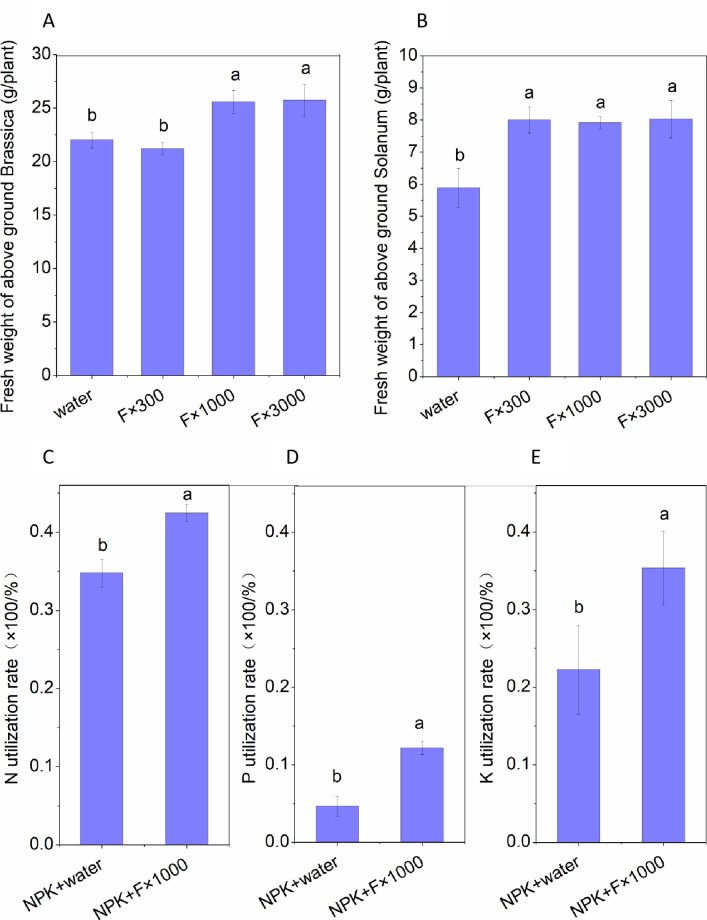
Effects of acetoin and 2,3-butanediol fermentation complex on vegetable growth and nutrient utilization efficiency. (**A**) Effect of different treatments on *B. rapa* yield. (**B**) Effect of different treatments on *S. lycopersicum* var. yield. Water: Control; F×300, F×1,000, F×3,000: acetoin and 2,3-butanediol fermentation product diluted 300 times, 1,000 times, and 3,000 times. (**C–E**) Effect of different treatments on nutrient utilization efficiency in *B. rapa*. Thirty days after *B. rapa* sowing, the utilization efficiency of nitrogen, phosphorus, and potassium was analyzed. Different lowercase letters on the bars indicate significant differences between treatments, while the same lowercase letters indicate no significant differences (*P* < 0.05).

Acetoin and 2,3-butanediol are microbial volatile compounds (28, 29) and have different volatilities. The effect of LB medium and water on *B. rapa* and *S. lycopersicum* var. culture was analyzed, the biomass was similar under the two conditions, and no statistical difference was observed. Therefore, vegetables treated with only water served as the control in the cultivation experiment. The fermentation mixture was diluted 300 (F×300), 1,000 (F×1,000), and 3,000 (F×3,000) times and then added to the culture of vegetables. Compared to the water-treated control, the fresh weight of the aboveground parts of *B. rapa* increased by 16.2% and 16.9% after treatment with F×1,000 and F×3,000, respectively, both reaching a significant level (Fig. 2A). However, the biomass slightly decreased with F×300 treatment. In *S. lycopersicum* var. cultivation experiment, compared to the water-treated control, the fresh weight of the aboveground parts increased by 36.1%, 34.7%, and 36.6% after the application of three different concentrations (F×300, F×1,000, and F×3,000) of fermentation mixture, all reaching a significant level (Fig. 2B). These results indicate that proper concentration of acetoin and 2,3-butanediol fermentation mixture significantly promote the growth of both *B. rapa* and *S. lycopersicum* var. under the same cultivation conditions. Since the acetoin and 2,3-butanediol fermentation mixture diluted 1,000 times exhibited the best growth promotion effect, this concentration was chosen for the subsequent experiments.

### Effect of acetoin and 2,3-butanediol fermentation mixture on nutrient utilization of *B. rapa*

Based on the promotion of acetoin and 2,3-butanediol mixture on vegetable growth, the nutrient utilization by *B. rape* was further analyzed. Cultivation experiments were carried out under different nutrient conditions, and the absorption of nitrogen, phosphorus, and potassium was measured. Compared to the control, the addition of the 1,000 times diluted acetoin and 2,3-butanediol fermentation mixture resulted in increased nutrient utilization efficiency for nitrogen (Fig. 2C), phosphorus (P_2_O_5_) (Fig. 2D), and potassium (K_2_O) (Fig. 2E) by 22.7%, 168.5%, and 64.6%, respectively. The results indicate that the acetoin and 2,3-butanediol fermentation mixture enhanced the utilization of nitrogen, phosphorus, and potassium in *B. rape* cultivation.

### Metagenomic analysis of rhizosphere soil in *B. rapa* cultivation

The cultivation experiments demonstrated that the acetoin and 2,3-butanediol fermentation mixture promoted the growth and increased the biomass of two vegetables. To elucidate the underlying mechanism of this promotion, metagenomic analysis of *B. rapa* rhizosphere soil treated with fermentation mixture and water was analyzed, aiming to explore the changes in the microbial community and functional genes in the rhizosphere.

The alpha diversity of *B. rapa* rhizosphere microbial community is shown in Table S3. The Chao1 index of the acetoin and 2,3-butanediol fermentation mixture treatment group was slightly higher than that of the water control group, but no significant difference was observed. There were no significant differences in the Shannon and Simpson indices between the treatment and control groups, suggesting that the community distribution was consistent under both conditions. Furthermore, the coverage values for both groups were 1.00, indicating that the sequencing fully covered the species in the community. Principal component analysis (PCA) results revealed that the intra-group samples of both the control and treatment groups clustered closely, indicating that the microbial community structure was stable within each group. Clear separation was observed between the two groups (Fig. S2A). Principal coordinates analysis (PCoA) also showed tight clustering of samples within each group, with minimal within-group differences, while the treatment and control groups were clearly separated in the PCoA space, indicating a significant difference in the microbial community composition between the rhizosphere soils of the two groups. Both PCA and PCoA analyses indicate that the acetoin and 2,3-butanediol fermentation mixture significantly impacted the microbial community structure, while the internal community structure of each group remained stable (Fig. S2B).

**Fig 3:**
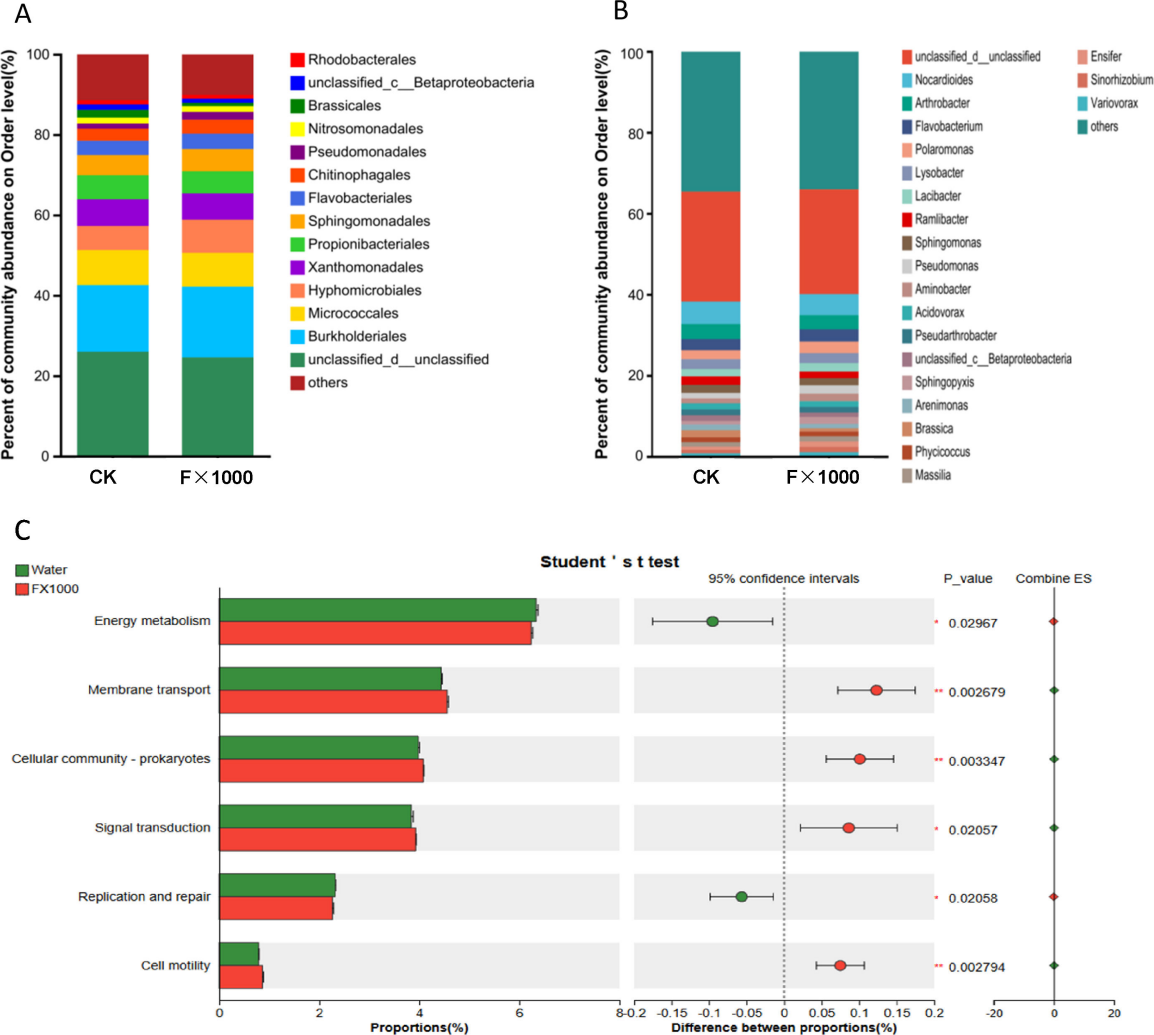
Effect of acetoin and 2,3-butanediol complex on the rhizosphere microbiome and their metabolic pathways of *B. rapa*. (**A**) Composition and abundance distribution of rhizosphere soil microbiome at the order level. (**B**) Composition and abundance distribution of rhizosphere soil microbiome at the genus level. (**C**) The metabolic pathways of the rhizosphere soil microbiome in *B. rapa*. The vertical axis represents the metabolic pathways of the rhizosphere soil microbiome, and the horizontal axis represents the percentage abundance of the metabolic pathways. Water represents the control with; F×1,000 represents the fermentation product of acetoin and 2,3-butanediol diluted 1,000 times. *, 0.01 < *P* ≤ 0.05; **, 0.001 < *P* ≤ 0.01; ***, P ≤ 0.001.

Based on the rhizosphere soil metagenome, the community composition and differences at the order and genus levels were analyzed. At the order level (Fig. 3A), the microbial communities of the F×1,000 treatment group and the control group (CK) were similar, though with different abundances. Microbial groups such as *Unclassified*, *Burkholderiales*, and *Xanthomonadales* had higher abundances, while *Flavobacteriales*, *Pseudomonadales*, and *Nitrosomonadales* had lower abundances, with no significant differences between the treatment and control groups. In the treatment group, the abundance of *Micrococcales* slightly decreased, whereas the abundances of *Hyphomicrobiales*, *Sphingomonadales*, and *Rhodobacterales* increased. At the genus level (Fig. 3B), unclassified species accounted for 25.86% of the total abundance. Additionally, species with an abundance of <0.01% (non-dominant species) were combined into the “others” category, representing approximately 33.99% of the total abundance. Table S4 lists 109 microbial genera that exhibited significant differences (*P* < 0.05) between the treatment and control groups. The genera *Nocardioides* and *Arthrobacter* had high abundances in both groups, suggesting they are dominant microbes in the rhizosphere, potentially involved in organic matter decomposition. Among the higher-abundance microbes, nine genera, including *Ensifer*, *Sinorhizobium*, *Variovorax*, *Polaromonas*, *Pseudomonas*, *Aminobacter*, *Cupriavidus*, *Bosea*, and *Shinella*, significantly increased in abundance in the treatment group compared to the control. Meanwhile, the abundance of *Nocardioides*, *Arthrobacter*, *unclassified_c__Betaproteobacteria*, *Arenimonas*, *Brassica*, *Ramilbacter*, *Bradyrhizobium*, and *Cellvibrio* significantly decreased. There was no significant difference in the abundance of 11 genera, including *Unclassified*, *Flavobacterium*, *Lysobacter*, *Lacibacter*, *Ramilbacter*, *Sphingomonas*, *Acidovorax*, *Pseudarthrobacter*, *Sphingopyxis*, *Phycicoccus*, and *Massilia*.

Genera such as *Ensifer*, *Sinorhizobium*, *Bosea*, and *Shinella* are nitrogen-fixing bacteria capable of converting atmospheric nitrogen (N₂) into plant-available ammonium (NH_4_^+^), particularly beneficial in nitrogen-deficient soils (30). *Pseudomonas* and *Aminobacter* can secrete organic acids that solubilize insoluble phosphate in the soil, enhancing phosphorus and potassium absorption by plants (31). These microbes are also applied in disease prevention and plant growth promotion (32). *Polaromonas* may produce chelating agents that enhance the absorption of trace metal elements (33). Additionally, *Pseudomonas*, *Variovorax*, *Shinella*, and *Bosea* can secrete indole-3-acetic acid (IAA) and other plant hormones that promote root growth and nutrient absorption (34–36). *Cupriavidus* and *Shinella* can secrete antioxidant enzymes (e.g., superoxide dismutase, catalase) to help plants eliminate reactive oxygen species (ROS), alleviating salt stress, drought, or other environmental stresses. *Cupriavidus* can also accumulate polyhydroxyalkanoates (PHA), which have potential benefits for plant growth (37). Overall, microbes whose abundance increased significantly can promote plant growth by providing nutrients, secreting hormones, enhancing stress resistance, and improving the rhizosphere microenvironment.

The microbes whose abundance decreased under treatment were primarily involved in organic matter degradation and soil organic carbon cycling, including *Nocardioides*, *Arenimonas*, *Cellvibrio*, *Ramilbacter*, and *unclassified_c__Betaproteobacteria*. This decrease suggests that the organic matter in the rhizosphere soil may have decreased following treatment. *Brassica*, a genus associated with cruciferous plants (Brassicaceae), exhibited the most significant reduction in abundance, warranting further analysis (38). *Bradyrhizobium*, a root-nodule nitrogen-fixing bacterium, showed a slight reduction under treatment, but this was not statistically significant (39). Although *Ramilbacter* (which can produce organic acids and promote phosphorus and potassium absorption) decreased in abundance, other microbes with similar functions, such as *Pseudomonas* and *Aminobacter*, increased significantly.

In addition to the genera with higher abundance mentioned above, 92 genera with lower abundance also exhibited significant changes (Table S5). Among them, 39 genera had significantly increased abundance in the treatment group, while 53 genera had significantly decreased abundance. Based on microbial function, the 39 genera with increased abundance mainly fall into three categories: promoting plant growth, enhancing environmental adaptability, and improving stress resistance, as well as some microbes with unknown function. Plant growth-promoting microbes can directly or indirectly enhance plant growth through nitrogen fixation, hormone secretion, or phosphate solubilization. For example, *Burkholderia* is a symbiotic bacterium that promotes plant growth by fixing nitrogen and secreting growth hormones (e.g., indole-3-acetic acid) (40). *Nitrosomonas* participates in ammonia oxidation, playing a key role in the nitrogen cycle and promoting soil nitrogen utilization. Microbes that enhance environmental adaptability, such as *Devosia*, which can degrade toxic substances, improve soil quality, and help plants grow in polluted environments (41); *Rhizobacter* may inhibit the growth of pathogenic microbes and enhance plant health (42); and *Knoellia*, which is related to soil microecological stability and indirectly increases plant disease resistance (43).

Among the 53 genera with decreased abundance, some are involved in the degradation of soil pollutants and may have direct or indirect effects on plant growth. Pollutant-degrading microbes include *Methylibium*, *Devosia*, *Sphingobium*, *Xanthomonas*, *Thermomonas*, *Mycobacterium*, and *Devosia*; nitrifying bacteria, such as *Nitrosomonas* and *Nitrososphaera*, convert ammonia to nitrite (NO_2_⁻). Other genera include seven microbes of unknown function, such as *unclassified_p__Acidobacteria*, *Methylotenera*, and *unclassified_o__Burkholderiales*. The metagenomic data were mapped to specific metabolic pathways using the KEGG database, and significant metabolic changes in rhizosphere microbes were observed. The differences in metabolic pathways are shown in Fig. 3C, with six significantly different functional pathways. Compared to the control, the treatment group showed significant increases in membrane transport, cellular community—prokaryotes, cell motility, and signal transduction. In contrast, the treatment group showed significantly lower abundances in energy metabolism and replication and repair functions.

### Nitrogen fixation-related genes in the rhizosphere soil of *B. rapa* cultivation

**Fig 4:**
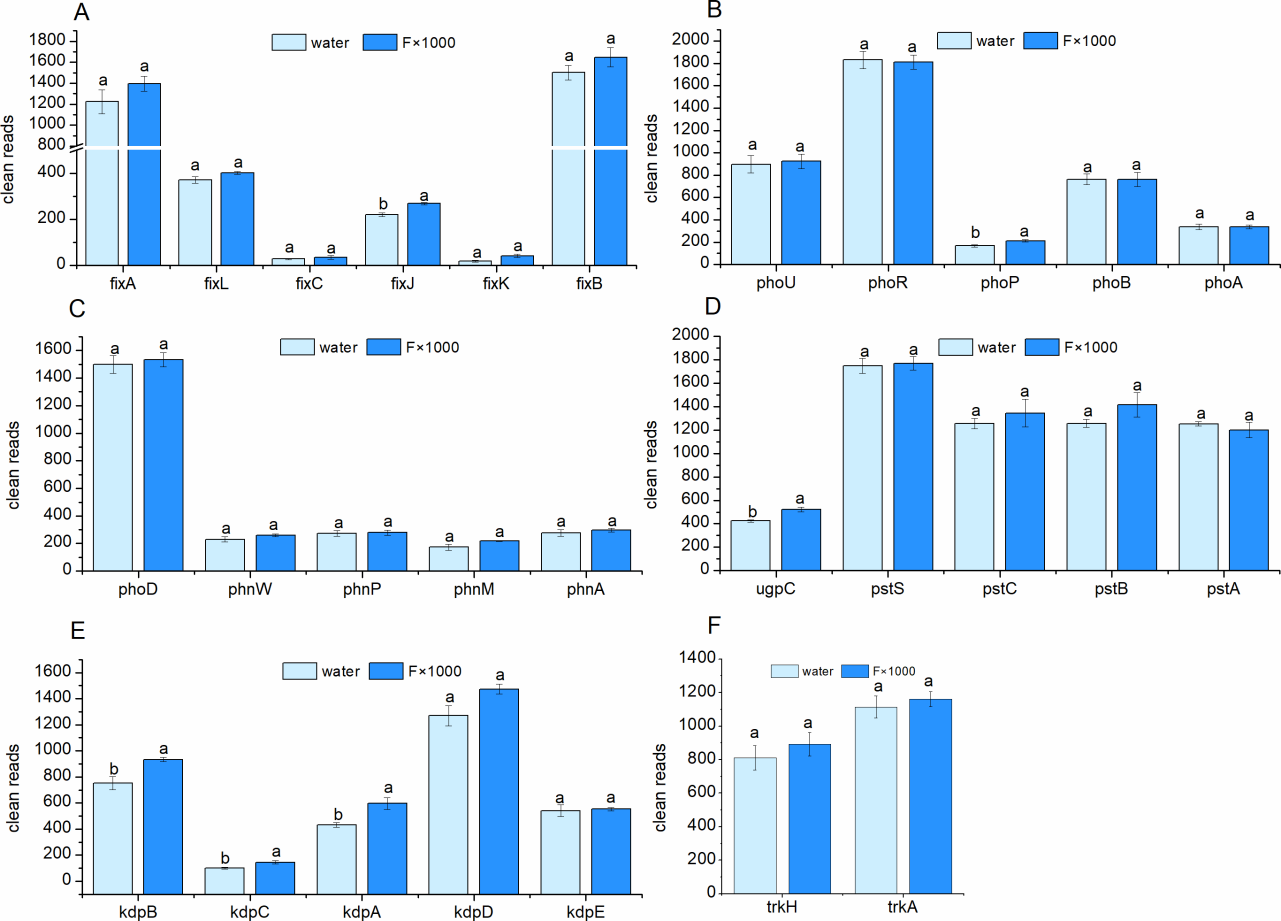
Effect of acetoin and 2,3-butanediol fermentation complex on nitrogen fixation, phosphorus, and potassium utilization-related genes in *B. rapa* rhizosphere microbiome. (**A**) Nitrogen fixation-related genes. (**B**) P-starvation response regulation-related genes. (**C**) Inorganic P solubilization and organic P-mineralization-related genes. (**D**) P-uptake and transport-related genes. (**E**) ATP-driven potassium transport system kdp operon-related genes. (**F**) Potassium transport system Trk-related genes. Water represents the control; F×1,000 represents the acetoin and 2,3-butanediol fermentation mixture diluted 1,000 times. Different lowercase letters indicate significant differences between treatments (*P* > 0.05), while the same lowercase letters indicate no significant differences between treatments (*P* < 0.05).

The *fix* gene cluster is a key genetic cluster in nitrogen-fixing microorganisms, mainly involved in the regulation of nitrogen fixation, low-oxygen adaptation, and electron transfer processes (44). The genes *fix*A/*fix*B encode the electron transfer complex, providing electrons for nitrogenase; *fix*C works with *fix*A/B to facilitate electron transfer; *fix*K encodes a transcription factor that activates the expression of various nitrogen fixation-related genes; *fix*J encodes a response regulator protein; and *fix*L encodes an oxygen-sensing histidine kinase, which detects changes in environmental oxygen levels. The *fix*LJ system, including *fix*J and *fix*L, regulates the transcription of downstream nitrogen fixation genes and plays a decisive role in whether bacteria initiate nitrogen fixation (45). Under both control and treatment conditions, the transcription levels of nitrogen fixation-related genes (*fix*A, *fix*L, *fix*C, *fix*J, *fix*K, and *fix*B) in the *fix* gene cluster were elevated, with the transcription of *fix*J significantly increased. The expression of *fix*A, *fix*L, and *fix*B was relatively high, while the expression of *fix*C and *fix*K was lower (Fig. 4A). These results indicate that the acetoin and 2,3-butanediol fermentation mixture enhanced the nitrogen fixation ability of rhizosphere soil microorganisms in *B. rapa* by promoting the transcription of nitrogen fixation-related genes in the fix gene cluster.

### Phosphorus utilization-related genes in rhizosphere soil of *B. rapa* cultivation

The soil metagenomic analysis suggests that the increase in the abundance of microorganisms such as *Pseudomonas* and *Aminobacter* may enhance the dissolution and absorption of insoluble phosphorus in the soil. Therefore, further analysis was conducted on genes potentially associated with plant phosphorus (P) uptake. Phosphorus is an essential element for plant growth and is often in a limiting state in natural environments. Microorganisms have evolved the P-starvation response regulation system to enhance their ability to acquire and utilize low concentrations of inorganic phosphorus (46). PhoA is alkaline phosphatase; PhoB is a response regulator in the Pho system, activating the transcription of phosphorus starvation response genes. PhoP regulates the expression of phosphorus starvation-related genes in response to low phosphorus signals, directly or indirectly regulating high-phosphorus transport genes and organic phosphorus metabolic enzymes (PhoA, PhoD), as well as the regulatory modulator PhoU. PhoR is a sensor kinase in the Pho system, which regulates the expression of the entire Pho regulon. In the treatment group, compared to the control group, the abundance of phosphorus starvation response regulation-related genes *pho*U (28) and *pho*P (47) increased, with *pho*P showing a significant increase (Fig. 4B). The transcription levels of other related genes remained relatively consistent. Genes such as *pho*D, *phn*W, *phn*P, *phn*M, and *phn*A are associated with inorganic phosphorus solubilization and organic phosphorus mineralization (48–51). PhoD encodes alkaline phosphatase, involved in organic phosphorus decomposition and the release of inorganic phosphorus, playing a key role in organic phosphorus mineralization. PhnW participates in the breakdown of phosphoalkylamine compounds, providing raw materials for phosphorus metabolism (49). PhnP is responsible for the degradation of organic phosphorus compounds, releasing available phosphates. PhnM catalyzes C-P bond cleavage, contributing to the utilization of organic phosphorus compounds. PhnA is related to phosphate absorption or transformation, possibly involved in the organic phosphorus mineralization pathway (52). After treatment, the abundance of inorganic P solubilization and organic P-mineralization related genes *pho*D (53), *phn*P, *phn*W (54), *phn*M (32), and *phn*A (29) increased, although not significantly (Fig. 4C). This indicates that volatile compounds treatment promotes the expression of genes related to inorganic phosphorus solubilization and organic phosphorus mineralization.

Genes such as *ugp*C, *pst*S, *pst*C, *pst*B, and *pst*A are associated with phosphorus uptake and transport (55). UgpC encodes the ATP-binding protein of the glycerol phosphate transport system, involved in energy metabolism and affecting the hydrolysis and release of pyrophosphate, possibly participating in the transport of phosphorylated ester compounds. The *pst*S encodes the phosphate-binding protein in the high-affinity phosphate transport system, responsible for capturing external phosphates. The *pst*C encodes the transmembrane transport protein in the *pst* system, closely related to phosphate transport across the membrane. The *pst*B encodes the ATP-binding protein in the *pst* system, providing energy for the active transport of phosphates (56). Compared to the control, the treatment group showed an increase in the abundance of genes related to phosphorus uptake and transport, including *ugp*C (57), *pst*S (57), *pst*C (57), and *pst*B (57) (Fig. 4D), with *ugp*C showing a significant increase. The upregulation of these genes after volatile compounds treatment indicates enhanced phosphorus uptake and transport. In conclusion, the treatment increased the abundance of genes related to phosphorus starvation response regulation, inorganic P solubilization, and organic P-mineralization, as well as P-uptake and transport, suggesting that volatile compounds promoted the mineralization and transport of phosphorus in soil, enhancing phosphorus absorption.

### Potassium utilization-related genes in rhizosphere soil of *B. rapa* cultivation

Potassium is one of the most important cations in bacterial cells, involved in maintaining osmotic pressure, pH stability, charge balance, and regulating various enzyme activities. To adapt to varying environmental potassium concentrations, microorganisms have evolved multiple potassium ion transport systems, with the *kdp* (58) and *trk* (59) gene clusters being the most classical two, each with complementary functions and different regulatory mechanisms. The *kdp* system operates under potassium deficiency, aiding survival in extreme environments, while the *trk* system functions under normal conditions to facilitate potassium ion absorption. The *kdp* gene cluster includes genes encoding transmembrane proteins (*kdp*A) responsible for potassium transport, catalytic subunits (*kdp*B) providing energy for ion transport, auxiliary subunits (*kdp*C) helping to stabilize the structure of kdpA/B complexes, and a two-component regulatory system (*kdp*D/*kdp*E) that senses external potassium concentrations and adjusts the expression of *kdp* genes (60). As shown in Fig. 4E, the expression of the *kdp* gene family members (*kdp*A, *kdp*B, and *kdp*C) in the treatment group was significantly higher than in the control group. The expression of *kdp*D in the treatment group was higher than in the control but did not reach statistical significance, while the expression of *kdp*E was similar between the two groups. This indicates that the acetoin and 2,3-butanediol fermentation mixture induced the transcription of *kdp*A, *kdp*B, *kdp*C, and *kdp*D, enhancing potassium active transport capacity. The *trk* gene cluster typically includes transmembrane protein genes (*trk*H) that form the potassium channel pore and cytoplasmic protein genes (*trk*A) that may regulate channel activity by binding with NAD(H). The *trk* system functions in normal or higher potassium concentration environments and is driven by membrane potential instead of ATP, maintaining the basic supply of potassium (59). As shown in Fig. 4F, compared to the control, the transcription levels of the *trk* gene family members (*trk*H and *trk*A) in the treatment group increased, though not significantly. These results suggest that the potassium transport function (*trk*) was enhanced in the acetoin and 2,3-butanediol fermentation mixture treatment group, promoting potassium transmembrane transport.

### Effect of acetoin and 2,3-butanediol fermentation mixture on salt stress tolerance of *B. rapa*

**Fig 5:**
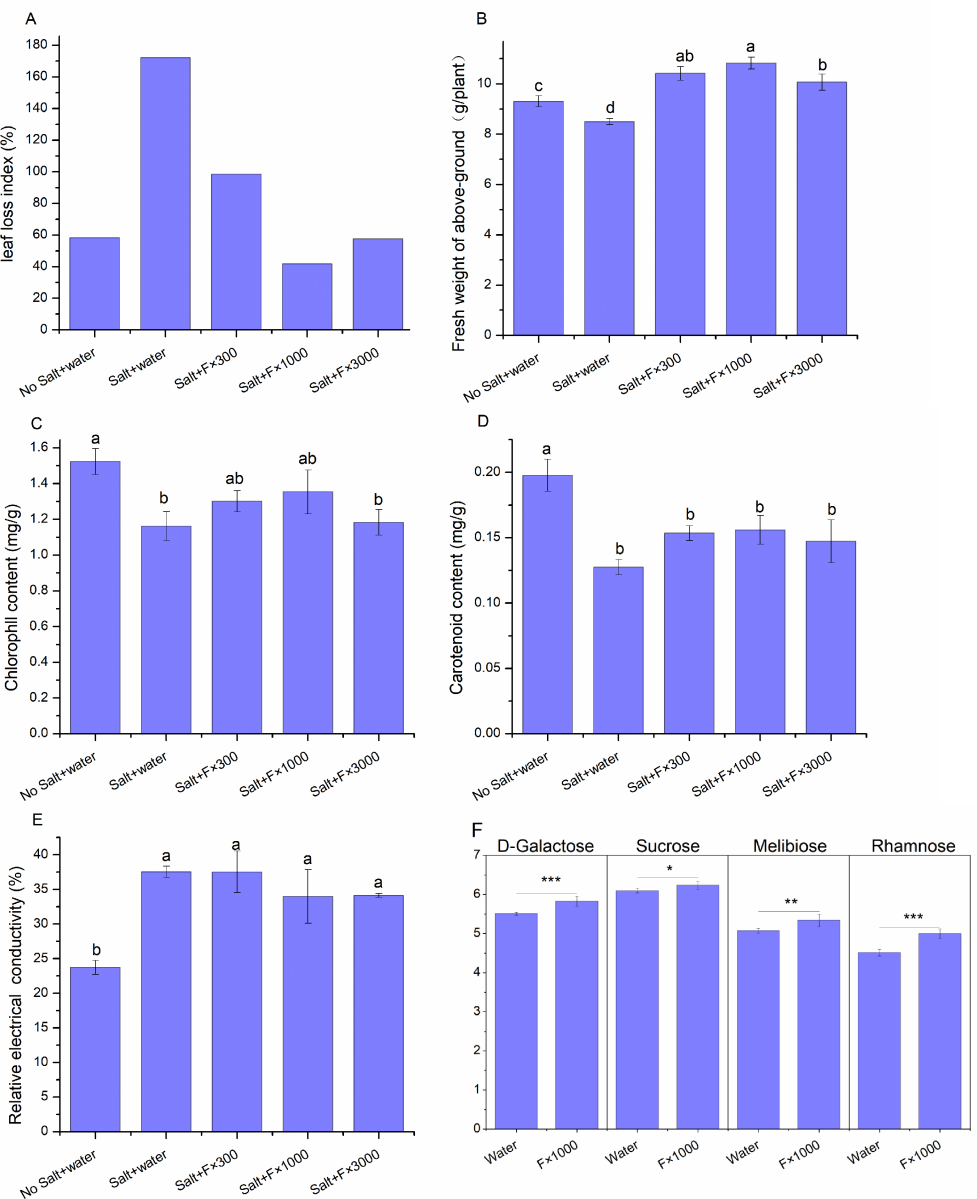
Effect of acetoin and 2,3-butanediol fermentation complex on salt tolerance in *B. rapa* and sugar content of *S. lycopersicum* var. leaf. (**A**) *B. rapa* leaf damage index under salt stress conditions. (**B**) Fresh weight of the aerial part of *B. rapa* under salt stress conditions. (**C**) Chlorophyll content in the aerial part of *B. rapa* under salt stress condition. (**D**) Carotenoid content in the aerial part of *B. rapa* under salt stress conditions. (**E**) Relative conductivity of the aerial part of *B. rapa* under salt stress conditions. No Salt + Water: negative control, no salt stress; Salt + Water: Salt stress; Salt + F × 300/Salt + F × 1,000/ Salt + F×3,000: Salt stress with fermentation mixture diluted 300, 1,000, and 3,000 times. Different lowercase letters indicate significant differences between treatments (*P* > 0.05), while the same lowercase letters indicate no significant differences between treatments (*P* < 0.05). (**F**) Effect of acetoin and 2,3-butanediol fermentation complex on sugar content in the leaf of *S. lycopersicum* var. Water: Control; F×1,000: acetoin and 2,3-butanediol fermentation mixture diluted 1,000 times. Significant differences are indicated by asterisks: *, 0.01 < *P* ≤ 0.05; **, 0.001 < *P* ≤ 0.01; ***, *P* ≤ 0.001.

Metagenomic analysis of rhizosphere soil revealed that the abundance of various microbial genera, such as *Cupriavidus* and *Shinella*, was significantly increased, and these microbes are known to enhance plant tolerance to salt, drought, and other stressors (37, 61). Therefore, the effect of acetoin and 2,3-butanediol complex on the growth of *B. rapa* under salt stress was studied. In the non-salt cultivation group, the leaf damage index of *B. rapa* was 58.33%. In contrast, the leaf damage index increased to 172.22% in the salt-stressed cultivation group. Under salt stress, after adding different concentrations (F×300, F×1,000, and F×3,000) of the fermentation mixture, the leaf damage indices of *B. rapa* were 98.51%, 41.79%, and 57.58%, respectively (Fig. 5A).

Additionally, the biomass of *B. rapa* under different cultivation conditions was compared. The biomass of the non-salt control group was 9.49% higher than in the salt-stressed group, with significant differences. In the treatment groups with different concentrations of volatile compounds, the biomass of *B. rapa* was 22.55%, 27.38%, and 18.48% higher than in the salt-stressed group, all reaching significant levels. These results suggest that the application of acetoin and 2,3-butanediol fermentation complex can effectively enhance *B. rapa*’s resistance to salt stress, with biomass even surpassing that of the non-salt stress control group (Fig. 5B).

The chlorophyll content in the non-salt control group was 31.17% higher than that in the salt-stressed group, which was statistically significant. After adding different concentrations (F×300, F×1,000, and F×3,000) of acetoin and 2,3-butanediol complex, the chlorophyll content was 12.01%, 16.53%, and 1.86% higher than that of the salt-stressed group, respectively (Fig. 5C). The carotenoid content in the non-salt control group was 55.14% higher than that in the salt-stressed group, with significant differences; after adding different concentrations of the acetoin and 2,3-butanediol complex, the carotenoid content was 20.47%, 22.35%, and 15.53% higher than that of the salt-stressed group (Fig. 5D). The analysis of chlorophyll and carotenoids indicates that salt stress inhibits *B. rapa*’s photosynthesis, but the volatile compounds can enhance photosynthesis under salt stress conditions, thus improving its ability to resist salt stress. The relative conductivity of *B. rapa* in the non-salt control group decreased by 36.77% compared to the salt-stressed group, which was statistically significant. In the treatment groups with different concentrations (F×300, F×1,000, and F×3,000) of volatile compounds, the relative conductivity was reduced by 0.03%, 9.47%, and 9.11%, respectively, compared to the salt-stressed group (Fig. 5E). Under salt stress, the permeability of the plant cell membrane increases, leading to an imbalance in the electrolyte equilibrium between the inside and outside of the cell, causing an increase in relative conductivity. The application of the acetoin and 2,3-butanediol fermentation mixture reduces relative conductivity under salt stress, thereby alleviating cellular damage caused by salt stress.

### Effect of acetoin and 2,3-butanediol fermentation mixture on non-targeted metabolomics of *S. lycopersicum* var.

**Fig 6:**
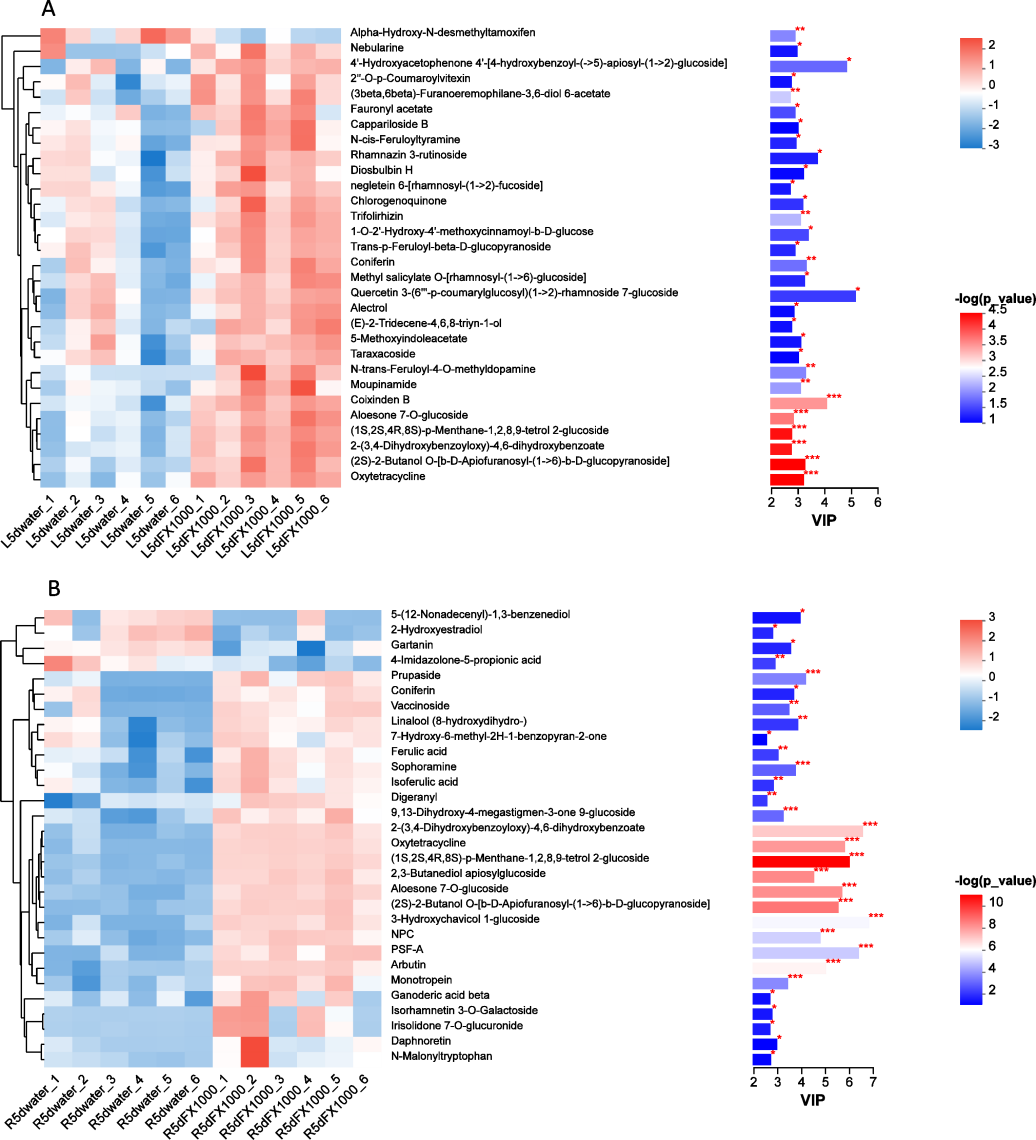
Effect of acetoin and 2,3-butanediol fermentation mixture on the metabolome of *S. lycopersicum* var. (**A**) Untargeted metabolome of leaf tissue. (**B**) Untargeted metabolome of root tissue. R5d water: Control with water; F×1,000: acetoin and 2,3-butanediol fermentation mixture diluted 1,000 times.

Since the acetoin and 2,3-butanediol fermentation mixture significantly promoted the growth of *S. lycopersicum* var., a further non-targeted metabolomic analysis was then conducted. As shown in Fig. 6, in the leaf tissues, the treatment group showed 142 significantly upregulated metabolites and 102 significantly downregulated metabolites compared to the control group. In the root tissues, the treatment group showed 128 significantly upregulated metabolites and 125 significantly downregulated metabolites. After treating with volatile compounds, compared to the control group, 67 metabolites were significantly upregulated in both the leaves and roots (Fig. S3). These upregulated metabolites can be divided into the following four categories. Carbohydrates and glycosides, including rhamnose, melibiose, sucrose, methyl salicylate O-[rhamnosyl-(1→6)-glucoside], are related to plant energy metabolism, signal transduction, and environmental adaptation (47, 53, 62). Phenolic compounds and derivatives, including 4-hydroxymellein, umbelliferone, and 4-*p*-coumaroylquinic acid, may have antioxidant, antibacterial, and stress resistance functions. Flavonoids and terpenoids, including trifolirhizin and gibberellin A38 glucosyl ester, may promote plant growth and stress tolerance. Nucleobases and nucleosides, including 2-hydroxyadenine and pseudouridine 5′-phosphate, are related to nucleic acid metabolism and plant stress adaptation. Other metabolites, such as oxytetracycline and butyric acid, are associated with antimicrobial activity (63, 64).

The clustering analysis of the top 30 significant differential metabolites in the leaves and roots is shown in Fig. 6A. In the leaf tissues, eight significantly upregulated metabolites were identified, including oxytetracycline, (2S)−2-butanol O-(β-D-apiofuranosyl-(1→6)-β-D-glucopyranoside), 2-(3,4-dihydroxybenzoyloxy)−4,6-dihydroxybenzoate, (1S,2S,4R,8S)-menthane-1,2,8,9-tetrol 2-glucoside, aloesone 7-O-glucoside, coxidine B, taraxacoside, and 5-methoxyindoleacetate. Oxytetracycline, with antimicrobial activity, may inhibit the growth of pathogenic bacteria (64); the significant upregulation in the F×1,000 treatment group suggests enhanced disease resistance. (2S)−2-butanol O-(β-D-apiofuranosyl-(1→6)-β-D-glucopyranoside) is a secondary metabolite potentially associated with stress response and antioxidant activity; its upregulation may enhance stress adaptability (65). 2-(3,4-dihydroxybenzoyloxy)−4,6-dihydroxybenzoate is a phenolic compound with antioxidant and free radical scavenging abilities; the treatment likely activated antioxidant mechanisms, helping plants alleviate oxidative stress (66). (1S,2S,4R,8S)-menthane-1,2,8,9-tetrol 2-glucoside is a glycosylated secondary metabolite involved in signaling during stress responses, and its upregulation reflects the treatment’s impact on glycosylation regulation, enhancing metabolic stability (67). Aloesone 7-O-glucoside, a glycoside, is related to antimicrobial and anti-inflammatory activities, and its upregulation may enhance plant defense against environmental stresses or pathogens (68). Coxidine B, a terpenoid compound, is associated with antioxidant properties and plant growth regulation; its significant upregulation suggests that treatment promotes growth or stress adaptation. Taraxacoside, a flavonoid, has antibacterial and antioxidant properties; its accumulation likely contributes to the regulation of the cell’s antioxidant defense (69, 70). The indole compound 5-methoxyindoleacetate may be involved in growth regulation (e.g., as an IAA-like compound) or stress responses (71); its upregulation is likely related to growth regulation and stress responses.

In the root tissues, clustering analysis of the top 30 significant differential metabolites revealed six upregulated compounds, including 2-(3,4-dihydroxybenzoxy)−4,6-dihydroxybenzoate, oxytetracycline, (1S,2S,4R,8S)-p-menthane-1,2,8,9-tetrol 2-glucoside, 2,3-butanediol apiosylglucoside, aloesone 7-O-glucoside, and (2S)−2-butanol O-[β-D-apiofuranosyl-(1→6)-β-D-glucopyranoside]. Compared to the leaf tissues, 2,3-butanediol apiosylglucoside was uniquely present in the roots and may help the plant cope with drought, high salt, and other environmental stresses by regulating osmotic balance. Sugar metabolites, such as melibiose, sucrose, D-galactose, and rhamnose, are associated with plant energy metabolism, signal transduction, and environmental adaptation (47, 53, 62). Compared to the control, the treatment significantly increased the abundance of these four sugars in the leaf (Fig. 5F), suggesting enhanced sugar metabolism or synthesis. Melibiose and sucrose are involved not only in plant growth and development but also in the response to various abiotic stresses, thereby improving the plant’s adaptation to abiotic stress. D-galactose, associated with antioxidant metabolism and the cell wall, supports plant growth and defense. Rhamnose, involved in secondary metabolism and strengthening the cell wall, enhances plant disease resistance and stress tolerance (62). In conclusion, the significant metabolites identified in the leaves and roots mainly include plant secondary metabolites and sugars, which may play roles in plant disease defense, antioxidant activity, free radical scavenging, and stress resistance.

### KEGG pathway enrichment analysis of leaf and root tissues of *S. lycopersicum* var.

Compared to the control group, the differential expression of genes in the leaves treated with acetoin and 2,3-butanediol complex was enriched in various metabolic pathways (Fig. S4A). After the treatment, the enriched pathways in the leaves included galactose metabolism, tryptophan metabolism, tyrosine metabolism, phenylpropanoid biosynthesis, and starch and sucrose metabolism. Phenylpropanoid biosynthesis is related to secondary metabolism and is responsible for the synthesis of stress-resistant substances such as lignin and phenolic compounds. The significant changes in this pathway may be associated with enhanced stress responses or defense mechanisms in plants. Starch and sucrose metabolism is involved in carbohydrate storage and breakdown and regulates cell growth and tolerance to salt and alkaline stresses. Galactose metabolism is involved in the metabolism and conversion of galactose, providing energy or carbon skeletons to plants, and may be related to cell wall synthesis or signaling molecules (72). Tryptophan metabolism and tyrosine metabolism are involved in the synthesis of auxins (IAA) and are related to plant growth, hormone synthesis, secondary metabolism, and stress responses.

Compared to the control group, the enriched pathways in the roots included alpha-linolenic acid metabolism, arachidonic acid metabolism, TCA cycle, ascorbate and aldarate metabolism, phenylpropanoid biosynthesis, plant hormone signal transduction, diterpenoid biosynthesis, and monoterpenoid biosynthesis (Fig. S4B). The enrichment of the alpha-linolenic acid metabolism pathway was the highest, with metabolic products of α-linolenic acid playing an important role in regulating membrane fluidity, plant signal transduction (such as jasmonic acid signaling pathways), and stress resistance. Arachidonic acid metabolism may be involved in the synthesis of signaling molecules, and after treatment, it may affect membrane stability or signal transduction by regulating arachidonic acid metabolism. Pathways such as the TCA cycle, ascorbate and aldarate metabolism, and phenylpropanoid biosynthesis were also significantly enriched, which are associated with enhanced energy metabolism, antioxidant response, and stress resistance in plants. Additionally, pathways like plant hormone signal transduction, diterpenoid biosynthesis, and monoterpenoid biosynthesis were also enriched, suggesting that volatile compounds might influence plant hormone levels, thereby regulating plant physiological and developmental processes.

## DISCUSSION

### Enhanced production of acetoin and 2,3-butanediol by metabolic engineering of *B. subtilis* AC-6

In previous work, *B. subtilis* AC-6 was selected from soil and mutated to produce a high concentration of acetoin and 2,3-butanediol. To analyze its genetic background, the whole genome of *B. subtilis* AC-6 was sequenced, and its metabolic pathway for the synthesis of acetoin and 2,3-butanediol was analyzed. The genome analysis revealed that *B. subtilis* AC-6 carries genes associated with acetoin degradation and acetate production pathways, including acetoin dehydrogenase, phosphate acetyltransferase, and diacetyl reductase. Consistent with fermentation results, acetate was identified as the major by-product during *B. subtilis* AC-6 fermentation. Although acetaldehyde was not detected, it is likely oxidized to acetate under aerobic conditions. Previous studies also demonstrated that acetoin could serve as an alternative carbon source when glucose was nearly depleted (26). Based on these findings, metabolic engineering strategies were applied to block acetoin degradation and acetate synthesis pathways. Specifically, deletion of the *acoA* gene encoding acetoin dehydrogenase subunit alpha and the *eutD* gene encoding phosphate acetyltransferase led to enhanced acetoin and 2,3-butanediol accumulation. Compared to the wild-type strain Bac01, the engineered strain Bac03 (Δ*acoA*, Δ*eutD*) exhibited 71.52% and 37.19% higher acetoin and 2,3-butanediol production, respectively, with almost no detectable acetate. Furthermore, overexpression of key rate-limiting enzyme genes (*alsS*, *alsD*, and *bdhA*) involved in acetoin and 2,3-butanediol biosynthesis, combined with optimized fermentation conditions, increased the titers of acetoin and 2,3-butanediol. Through integration of whole-genome analysis and metabolic pathway optimization, the production of acetoin and 2,3-butanediol by *B. subtilis* AC-6 was significantly improved.

### The addition of the acetoin and 2,3-butanediol fermentation mixture has the potential to reduce chemical fertilizer use

With the addition of acetoin and 2,3-butanediol fermentation complex, the cultivation of *B. rapa* and *S. lycopersicum* var. was conducted. The results show that the growth-promoting effect of the acetoin and 2,3-butanediol fermentation product on vegetables is not linearly related to the added concentration, and only the appropriate concentration can achieve the best growth-promoting effect. Based on the analysis of the growth promotion, the fertilizer nutrient utilization rates were also analyzed. In *B. rapa* cultivation, the addition of the acetoin and 2,3-butanediol fermentation complex led to increases of 22.7%, 168.5%, and 64.6% in nitrogen (N), phosphorus (P_2_O_5_), and potassium (K_2_O) utilization rates, respectively, and reached significant levels. In agricultural production, chemical fertilizers such as nitrogen, phosphorus, and potassium are widely used to increase crop yields and shorten growth cycles. There is growing attention to reducing the use of chemical fertilizers and improving their utilization efficiency. The significant increase in nutrient utilization rates in this study suggests that the acetoin and 2,3-butanediol fermentation products may offer potential to reduce chemical fertilizer application.

To explore the mechanism behind the growth promotion and nutrients utilization improvement, changes in the rhizosphere microbiome were analyzed. The results showed significant changes in the microbial community composition, abundance, metabolic pathways, and genes related to nitrogen, phosphorus, and potassium metabolism. The abundance of genera such as *Ensifer*, *Sinorhizobium*, *Bosea*, *Shinella*, and *Burkholderia* significantly increased in the volatile compounds treatment group, which are typically associated with nitrogen fixation in the rhizosphere, thereby enhancing soil nitrogen fixation capacity. Moreover, the abundance of *Nitrosomonas* increased significantly after volatile compounds mixture treatment, which is involved in ammonia oxidation and plays a crucial role in the nitrogen cycle, promoting nitrogen utilization in soil (73). The increase in nitrogen-fixing and ammonia-oxidizing microbes in the rapeseed rhizosphere soil was consistent with the enhanced nitrogen utilization rates. The increase in the abundance of *Aminobacter* and *Pseudomonas* genera (31) under volatile compounds treatment was also notable, as these genera are typically related to phosphorus and potassium absorption (73). They enhance nutrient uptake by secreting organic acids to solubilize insoluble phosphates in the soil, thereby increasing nutrient availability.

In addition to the enhancement of nitrogen, phosphorus, and potassium utilization, volatile compounds also led to an increase in the abundance of microbes that promote plant growth through multiple pathways, such as auxin secretion, enhancing stress resistance, and improving the rhizosphere microenvironment, which contributes to plant growth and stress tolerance (73–75). Analysis of the nitrogen fixation genes (fix gene cluster) in the rhizosphere microbiome of *B. rapa* showed increased transcription levels of all genes (*fix*A, *fix*L, *fix*C, *fix*J, *fix*K, and *fix*B), with a significant increase in the transcription of *fix*J. The *fix* gene cluster is crucial for nitrogen fixation in rhizobia and symbiotic nitrogen-fixing bacteria, maintaining and regulating nitrogenase activity.

Further analysis of genes related to phosphorus metabolism, such as those involved in P-starvation response regulation, inorganic P solubilization, organic P-mineralization, and P-uptake and transport, revealed that the relative abundance of genes such as *pho*U and *pho*P involved in phosphorus uptake increased significantly after the addition of the fermentation products. Additionally, genes related to inorganic P solubilization and organic P-mineralization (*pho*D, *phn*W, *phn*M, and *phn*A) (76, 77) showed increased abundance, suggesting enhanced phosphorus availability and absorption in *B. rapa*. In terms of potassium ion transport, the transcription levels of the *kdp* and *trk* gene families (74, 78) involved in potassium uptake and transport were increased following treatment with the fermentation products. Metagenomic analysis further revealed significant changes in the rhizosphere microbiome’s metabolic pathways after the addition of volatile compounds. Enhanced metabolic activities in the soil microbiome include membrane transport, signal transduction, and cellular movement, thereby improving adaptability, metabolic activity, and signal-regulation capabilities. These changes in membrane transport, nutrient uptake, and stress tolerance may have positive impacts on the vegetable’s ability to absorb nitrogen, phosphorus, and potassium from the soil and adapt to high salt stress.

### Improvement of salt stress tolerance by acetoin and 2,3-butanediol fermentation mixture

Analysis of rhizosphere soil microbiomes and KEGG metabolic pathway shows that the addition of volatile compounds can enhance vegetable tolerance to salt stresses. Therefore, further analysis of *B. rapa* growth under salt stress conditions was performed. When the concentration of sodium chloride in the soil was 7.5 mg/g dry weight, the leaf damage index of *B. rapa* was 2.95 times that of the non-salt-stressed control. After the addition of a 1,000-fold dilution of the volatile compounds, the leaf damage was 71.6% of that of the non-salt-stressed control. Under salt stress, the biomass of *B. rapa* decreased by 8.67%; however, after adding acetoin and 2,3-butanediol mixture at different dilution concentrations (F×300, F×1,000, and F×3,000), the yield of *B. rapa* increased by 11.9%, 16.3%, and 8.2%, respectively, compared to the non-salt-stressed control.

Under salt stress, the chlorophyll content of *B. rapa* decreased; after adding acetoin and 2,3-butanediol compounds at different concentrations (F×300, F×1,000, and F×3,000), the chlorophyll content increased by 12.01%, 16.53%, and 1.86%, respectively, compared to the salt-stressed environment. The trend of carotenoid content was similar to that of chlorophyll content. After adding acetoin and 2,3-butanediol mixture at different concentrations, the carotenoid content increased by 20.47%, 22.35%, and 15.53%, respectively, compared to the salt-stressed environment. Under salt stress, the electrical conductivity of *B. rapa* significantly increased. The relative electrical conductivity decreased by 9.47% when the volatile compounds (F×1,000) were added. These results indicate that the addition of acetoin and 2,3-butanediol compounds reduced the leaf damage index, increased chlorophyll and carotenoid content, lowered relative electrical conductivity, and significantly improved the *B. rapa* yield.

### Non-targeted metabolomics of *S. lycopersicum* var. revealed the acetoin and 2,3-butanediol fermentation mixture promoted plant stress resistance and hormone synthesis

The metabolic profiles of *S. lycopersicum* var. roots and leaves were analyzed with the addition of the acetoin and 2,3-butanediol mixture. Compared to the control, the metabolites with significantly upregulated abundance mainly included: carbohydrates and glycosides, phenolic compounds and their derivatives, flavonoids and terpenoid compounds, base and nucleoside-related metabolites, as well as other bioactive compounds. These upregulated metabolites contribute to energy storage, enhanced stress resistance, and growth regulation in plants.

Carbohydrates and glycosides are typically associated with plant energy storage metabolism, signal transduction, and environmental adaptation. The abundance of rhamnose, melibiose, D-galactose, and sucrose significantly increased, which could affect plant growth and development and enhance plant resistance to abiotic stresses, such as drought and high salinity, helping maintain cellular integrity (47, 53, 62, 72). Phenolic compounds and their derivatives, such as umbelliferone and 4-*p*-coumaroylquinic acid, may participate in antioxidant processes, improving environmental adaptability. Flavonoids and terpenoid compounds, such as trifolirhizin and gibberellin A38 glucosyl ester, play regulatory roles in plant growth or development. Aloesone 7-O-glucoside can inhibit the growth of various pathogens and reduce oxidative damage caused by environmental stress, possibly regulating metabolic pathways in plant stress adaptation (68). (2S)−2-butanol O-[β-D-apiofuranosyl-(1→6)-β-D-glucopyranoside] improves plant survival under high salt and drought stress by stabilizing proteins and cell membranes (65). Metabolic pathway enrichment analysis based on leaf and root metabolomes after acetoin and 2,3-butanediol treatment revealed that the enriched pathways in leaves mainly included secondary metabolism, carbon metabolism and energy, amino acid metabolism, and auxin biosynthesis. These enriched pathways are related to plant stress responses, signal molecules, and hormone synthesis (79).

In the roots, the main enriched pathways after volatile compounds treatment included lipid metabolism, secondary metabolite synthesis, energy and antioxidant metabolism, and plant hormone biosynthesis. Alpha-linolenic acid metabolism and arachidonic acid metabolism could affect cell membrane fluidity and signal molecule generation (80). Secondary metabolism of phenylpropanoid biosynthesis may regulate plant stress resistance (81). The TCA cycle and ascorbate and aldarate metabolism are related to energy supply and stress alleviation (82). Plant hormone signal transduction, diterpenoid biosynthesis, and monoterpenoid biosynthesis may be related to plant hormone synthesis and growth regulation (83–85). Based on the enrichment of metabolic pathways in both leaves and roots, it is evident that acetoin and 2,3-butanediol compounds enhanced metabolic pathways related to plant stress responses, energy metabolism, and hormone synthesis.

These findings suggest that acetoin and 2,3-butanediol compounds, as signal molecules, regulate the rhizosphere microbiome, metabolism, and plant–microbe interactions; the indigenous microbiota in the rhizosphere that promote nitrogen, phosphorus, and potassium absorption and enhance environmental stress resistance were recruited. This ultimately improves the plant’s utilization of nutrients like nitrogen, phosphorus, and potassium and enhances its tolerance to high salt stress. This study provides new ideas of recruiting and utilizing indigenous rhizosphere soil microbiota with volatile compounds to promote plant growth and improve tolerance to high salt stress environments, also offers new strategies for reducing the use of chemical fertilizers like nitrogen, phosphorus, and potassium in agricultural production.

### Conclusion

*Bacillus* volatile compounds of acetoin and 2,3-butanediol function as key signaling molecules coordinating rhizosphere microbial communities, plant metabolism, and plant–microbe interactions. High-level production of these volatiles enabled systematic evaluation of their ecological and physiological roles. The application of volatile compounds markedly enhanced plant growth, nutrient use efficiency, and salt stress tolerance in vegetables. Metagenomic analyses revealed substantial restructuring of the rhizosphere microbiome, with enrichment of microbial taxa and functional genes involved in nitrogen fixation, phosphorus and potassium solubilization, stress resistance, and plant growth promotion. Plant metabolomics further showed activation of pathways related to antioxidant defense, stress alleviation, and hormone-mediated growth regulation. Importantly, by enhancing the mobilization and biological utilization of soil nutrients, microbial volatiles reduce plant dependence on external chemical fertilizer inputs. These findings suggest that volatile-mediated activation of native soil microbiomes represents a promising, environmentally friendly strategy to reduce chemical fertilizer input and improve nutrient sustainability in agricultural systems.

## Data Availability

Sequencing data were deposited in the NCBI SRA under BioProject number PRJNA1182851.

## SUPPLEMENTAL MATERIAL

Supplemental material aem.02523-25-s0001.pdf Tables S1 to S5; Fig. S1 to S4.

ASM does not own the copyrights to Supplemental Material that may be linked to, or accessed through, an article. The authors have granted ASM a non-exclusive, world-wide license to publish the Supplemental Material files. Please contact the corresponding author directly for reuse.
