# Supplementary material for: Integrated metagenomic–metabolomic insights into plant–microbe interactions mediated by Bacillus volatile compounds
Source: Appl Environ Microbiol. 2026 Mar 30;92(4):e02523-25. doi: 10.1128/aem.02523-25 (PMC13101463; doi:10.1128/aem.02523-25)
Supplement: Supplemental material — Tables S1 to S5; Fig. S1 to S4. [file aem.02523-25-s0001.pdf]

Integrated metagenomic–metabolomic insights into plant–microbe interactions mediated by Bacillus volatile compounds

Haiqian Yang[1,2#], Wei Liu[4#], Jiwei Niu[4], Biao Geng[1,2], Pengfei Qiu[4], Hongshun Li[4], Junping Bao[4], Xin Pu[1,2], Yong Li[1,2], Xiaojing Jia[1,2], Yingxiang Sun[3]*, Yejun Han[1,2]*

[1]State Key Laboratory of Biopharmaceutical Preparation and Delivery, Institute of Process Engineering, Chinese Academy of Sciences, Beijing 100190, China.

[2]University of Chinese Academy of Sciences, Beijing 100049, PR China.

[3]Syngenta Group China, Sinofert Holdings Limited, Beijing, 100069, China.

[4]National Engineering Research Center for Cultivated Land Protection, Sinochem Agriculture Linyi R&D Center, Linyi, 276024, China.

#These authors contributed equally

Running Title: Volatile compounds regulate plant–microbe interactions

*Address correspondence to:

Yingxiang Sun yingxiang.sun@syngentagroup.cn

Yejun Han yjhan@ipe.ac.cn

Supplementary

Table S1.

Bacterial strains and plasmids applied in present study.

Strains	Description	Source
Escherichia coli Trans1-T1	Strain used for plasmid constructions	Transgen Biotech
Bacillus subtilis AC-6		
Bac	Wild-type strain, acetoin production	Lab collection
Bac01	Bac harboring the plasmid pMK4-P_{xylA}-comK	This study
Bac02	Bac01 derivate, knockout of the native *acoA* gene	This study
Bac03	Bac02 derivate, knockout of the native *eutD* gene	This study
Bac04	Bac03 harboring the plasmid pMA5-HpaII-alsS-HpaII-alsD-bdhA	This study
**Plasmids**		
pMK4-comk	Plasmid for expressing *comK* gene	Lab collection
pDG148-cre	Plasmid for expressing *cre* gene	Lab collection
pMD19T-aea-T7P	Plasmid having *ermC* gene	Lab collection
pMA5-HpaII-alsS-HpaII-alsD-bdhA	Plasmid for expressing *alsS* gene with P_{HpaII} promoter and *alsD-bdhA* gene cluster with P_{HpaII} promoter	This study

Table S2.

Genome characteristics of *B. subtilis* AC-6

**Characteristics**	**Values**
Total sequence length/bp	4051215
GC ratio/%	43.75
Max gene length	123927
Min gene length	1600
Average gene length/bp	845.40
Protein coding genes	4301
tRNA quantity	87
16S rRNA quantity	10

Table S3.

Alpha diversity of the rhizosphere soil microbial community in *B. rapa*

**Index**	**Water**	**F×1000**
Chao1	141.33±3.11[a]	145.33±4.44[a]
Shannon	4.04±0.01[a]	4.06±0.08[a]
Simpson	0.038±0.004[a]	0.036±0.010[a]
Coverage	1.00[a]	1.00[a]

*All data was shown as mean ± standard deviation (n=3). Superscript lowercase letters indicated the significant difference calculated by ANOVA at $P < 0.05$.

Table S4.

Comparative analysis of microbial community abundance at the genus level in the rhizosphere soil of *B. rapa*

Name	F×1000[a]-Mean(%)	Water[b]-Mean(%)	P-value
g__Nocardioides	5.977	6.852	0.02181
g__Arthrobacter	5.581	6.306	0.02663
g__Pseudomonas	3.88	2.552	0.00317
g__Aminobacter	3.25	2.241	4.63E-05
g__Polaromonas	2.022	1.691	0.004035
g__Brassica	1.405	3.391	0.0008347
g__Variovorax	1.21	0.7939	0.0002845
g__Ensifer	0.6457	0.3653	0.0001284
g__Cupriavidus	0.5517	0.2348	0.0001034
g__Ramlibacter	0.373	0.5664	0.0005972
g__Bosea	0.3409	0.2226	0.006228
g__Bradyrhizobium	0.3059	0.3584	0.03963
g__unclassified_c__Betaproteobacteria	0.2753	0.455	0.0002507
g__Arenimonas	0.2042	0.2797	0.003997
g__Shinella	0.1441	0.09079	0.02841
g__Methylibium	0.1217	0.103	0.02428
g__Knoellia	0.1168	0.09449	0.02988
g__Sinorhizobium	0.1022	0.07993	0.002302
g__Duganella	0.0908	0.05974	0.00361
g__Burkholderia	0.08532	0.06424	0.001156
g__Nitrosomonas	0.07556	0.06071	0.0111
g__unclassified_p__Acidobacteria	0.06779	0.09716	0.01292
g__Methylotenera	0.06125	0.07931	0.03924

g__Cellvibrio	0.05906	0.2626	0.002548
g__Devosia	0.04974	0.06675	0.009318
g__Rhizobacter	0.04681	0.02604	0.04537
g__Xanthomonas	0.04482	0.05767	0.04841
g__unclassified_p__Gemmatimonadetes	0.04171	0.05231	0.04343
g__Thermomonas	0.0358	0.0454	0.008971
g__Exaiptasia	0.03178	0.06641	0.0005017
g__unclassified_p__Candidatus_Rokubacteria	0.02775	0.03719	0.03272
g__Aeromicrobium	0.02737	0.03449	0.03629
g__Zea	0.02487	0.1312	6.57E-05
g__Mycobacterium	0.02447	0.06871	0.001263
g__Novosphingobium	0.02015	0.02736	0.03773
g__Woodruffvirus	0.019	0.00222	0.0003418
g__Mytilus	0.0181	0.03699	0.001712
g__unclassified_p__Spirochaetes	0.017	0.01289	0.003317
g__Qipengyuania	0.0153	0.009405	0.04787
g__Arabidopsis	0.01464	0.04245	0.001022
g__Aquabacterium	0.01458	0.01987	0.007129
g__Orbicella	0.01402	0.02387	0.001111
g__Sinapis	0.0137	0.03522	0.004432
g__Micromonospora	0.01355	0.01727	0.0072
g__Sphingobium	0.01296	0.009601	0.005309
g__unclassified_f__Myoviridae	0.01204	0.006994	0.01605
g__Nitrososphaera	0.01115	0.01413	0.01586
g__unclassified_o__Burkholderiales	0.008522	0.006056	0.04089
g__Aromatoleum	0.007731	0.003781	0.005814
g__Raphanus	0.00671	0.01754	0.01032

g__Acinetobacter	0.005256	0.0108	0.001925
g__Nitzschia	0.004474	0.0527	0.01745
g__Microthlaspi	0.004131	0.00876	0.002793
g__Corallococcus	0.003181	0.00972	0.02503
g__Rariglobus	0.002712	0.00568	0.02014
g__Tardibacter	0.002468	0.008065	0.005471
g__Helsingorvirus	0.002249	0.003609	0.04541
g__Brenneria	0.002128	0.0004734	0.02339
g__Euzebya	0.001924	0.001115	0.04882
g__Microbotryum	0.001748	0.0004371	0.003753
g__Herminiimonas	0.001743	0.0004304	0.004304
g__Usitatibacter	0.001374	0.0005883	0.008931
g__unclassified_o__Bdellovibrionales	0.001373	0	0.0002883
g__unclassified_f__Sinobacteraceae	0.00129	0.0001002	0.002208
g__Pseudorivibacter	0.00128	0.001884	0.04101
g__Porphyridium	0.001138	0.0001445	0.02536
g__Immanueltrevirus	0.001085	9.90E-05	0.04076
g__Olpidium	0.0009778	5.35E-05	0.002019
g__Leptolyngbya	0.0009381	0.001404	0.005436
g__Nitratireductor	0.0008981	0.0005306	0.02235
g__Enterobacter	0.0008631	0.006018	0.0003257
g__Candidatus_Dormibacter	0.0008577	0.0001002	0.0002744
g__Blyttiomyces	0.0008574	0.0001445	0.006818
g__Papaver	0.0008402	4.55E-05	0.03264
g__Rothia	0.0007367	0.001843	0.03306
g__Ficleduovirus	0.0006354	0.0001912	0.02968
g__Geminisphaera	0.0005514	0.0001457	0.01234
g__Hibiscus	0.0004753	0.002139	0.0005555

g__Xylophilus	0.0004316	0.001208	0.05052
g__Ochrobactrum	0.0004313	9.10E-05	0.03192
g__Trametes	0.0003819	0	0.03778
g__Siphonobacter	0.0003782	0	0.04927
g__Aestuariisphingobium	0.0003691	0.001207	0.04617
g__Allomyces	0.0003421	4.55E-05	0.007736
g__unclassified_p__Cyanobacteria	0.0003418	0.001182	0.04652
g__Micrococcus	0.0003379	4.55E-05	0.02589
g__Leifsonia	0.0003376	0	0.03209
g__Segeticoccus	0.0003291	0.001251	0.01218
g__Yuavirus	0.0003066	0	0.03027
g__Ruegeria	0.0002978	0	0.0006783
g__unclassified_o__Desulfobacterales	0.0002581	0	0.02966
g__Clostridioides	0.0002578	0.0009824	0.014
g__Enhygromyxa	0.0002223	0.001124	0.02261
g__unclassified_o__Hyphomicrobiales	0.000222	0.001035	0.02615
g__Rhodocytophaga	0.0002175	0.000635	0.0446
g__Kineococcus	0.0001732	0.0007151	0.02377
g__unclassified_f__Phycodnaviridae	0.0001289	0	8.05E-06
g__Methylophaga	8.92E-05	0.001056	0.04201
g__unclassified_p__Candidatus_Fermentibacteria	8.89E-05	0.0006375	0.01205
g__Thalassiosira	4.46E-05	0.0008048	0.01414
g__Aphanomyces	4.46E-05	0.0004814	0.02459
g__unclassified_o__Pseudomonadales	4.43E-05	0.001948	0.0002596
g__Obolenskvirus	4.43E-05	0.0002459	0.04753

g__Fluviicola	4.00E-05	0.0007151	0.01102
g__Oleiharenicola	0	0.0002447	0.01058
g__Escherichia	0	0.0002447	0.01058
g__unclassified_c__Verrucomicrobiae	0	0.0002459	0.01173
g__Roseburia	0	0.0001992	0.03066
g__unclassified_c__Chloroflexia	0	0.0004692	0.0433

[a] Treating with acetoin and 2,3-butanediol fermentation mixture diluted 1000 times.

[b] Treating with water as a control.

Table S5.

Metabolites significantly upregulated in *S. lycopersicum* var. treated with an acetoin and 2,3-butanediol fermentation mixture[a]

#	Metabolites	#	Metabolites	#	Metabolites
1[b]	Rhamnose	24	Benzyl gentiobioside	47	Kelampayoside A
2	(2S)-2-Butanol O-[b-D-Apiofuranosyl-(1->6)-b-D-glucopyranoside]	25	4'-Hydroxyacetophenone 4'-[4-hydroxybenzoyl-(->5)-apiosyl-(1->2)-glucoside]	48	negletein 6-[rhamnosyl-(1->2)-fucoside]
3	Oxytetracycline	26	Pseudouridine 5'-phosphate	49	3',6-Disinapoylsucrose
4	(1S,2S,4R,8S)-p-Menthane-1,2,8,9-tetrol 2-glucoside	27	Herierin III	50	(E)-2-Tridecene-4,6,8-triyn-1-ol
5	2-(3,4-Dihydroxybenzoyloxy)-4,6-dihydroxybenzoate	28	Gibberellin A38 glucosyl ester	51	4-Methoxybenzyl glucoside
6	2-Hydroxyadenine	29	Sucrose	52	Geranyl arabinopyranosyl-glucoside
7	Aloesone 7-O-glucoside	30	Kievitone	53	(+/-)-threo-1-(p-Hydroxyphenyl)propylene glycol 4'-glucoside
8	Dihydromethysticin	31	1-O-2'-Hydroxy-4'-methoxycinnamoyl-b-D-glucose	54	11-Deacetylvaltrate 11-(3-hydroxy-3-methylbutanoate)
9	Isoeugenitin	32	Aspirin	55	4-p-Coumaroylquinic acid
10	Coniferyl Aldehyde	33	N-Caffeoyltryptophan	56	Zingerone glucoside
11	N-Acetylputrescine	34	Zizybeoside I	57	Taraxacoside
12	(3beta,6beta)-Furanoeremophilane-3,6-diol 6-acetate	35	Tridecanoic acid	58	25-Acetylvulgaroside
13	Monotropein	36	Isoxanthohumol B	59	Cappariloside B

14	Melibiose	37	Chlorogenoquinone	60	3,4-Dihydro-2H-1-benzopyran-2-one
15	Trifolirhizin	38	Cyclopassifloic acid E	61	Butyric acid
16	Moupinamide	39	Alpha-Tocotrienol	62	3-Hydroxyanthranilic Acid
17	Hordenine	40	3-Methoxytyramine	63	5-Hydroxy-2,3-dimethyl-1,4-naphthoquinone
18	N-trans-Feruloyl-4-O-methyldopamine	41	Trans-p-Feruloyl-beta-D-glucopyranoside	64	3-O-p-Coumaroylquinic acid
19	4-Hydroxymellein	42	Methyl salicylate O-[rhamnosyl-(1->6)-glucoside]	65	Oxalic acid dibutyl ester
20	5,7-Dihydroxy-4'-methoxy-8-methylflavanone	43	Epiacoronene	66	Dehydrooreadone
21	Coniferin	44	Umbelliferone	67	Homomethionine
22	Cis-Zeatin O-glucoside	45	Rhamnazin 3-rutinoside		
23	2-Hydroxyestradiol	46	2-Acetyl-3,6-dimethylpyrazine		

[a] Acetoin and 2,3-butanediol fermentation mixture was diluted 1000 times; water as used as a control.

[b] Ranked by ascending *p*-values.

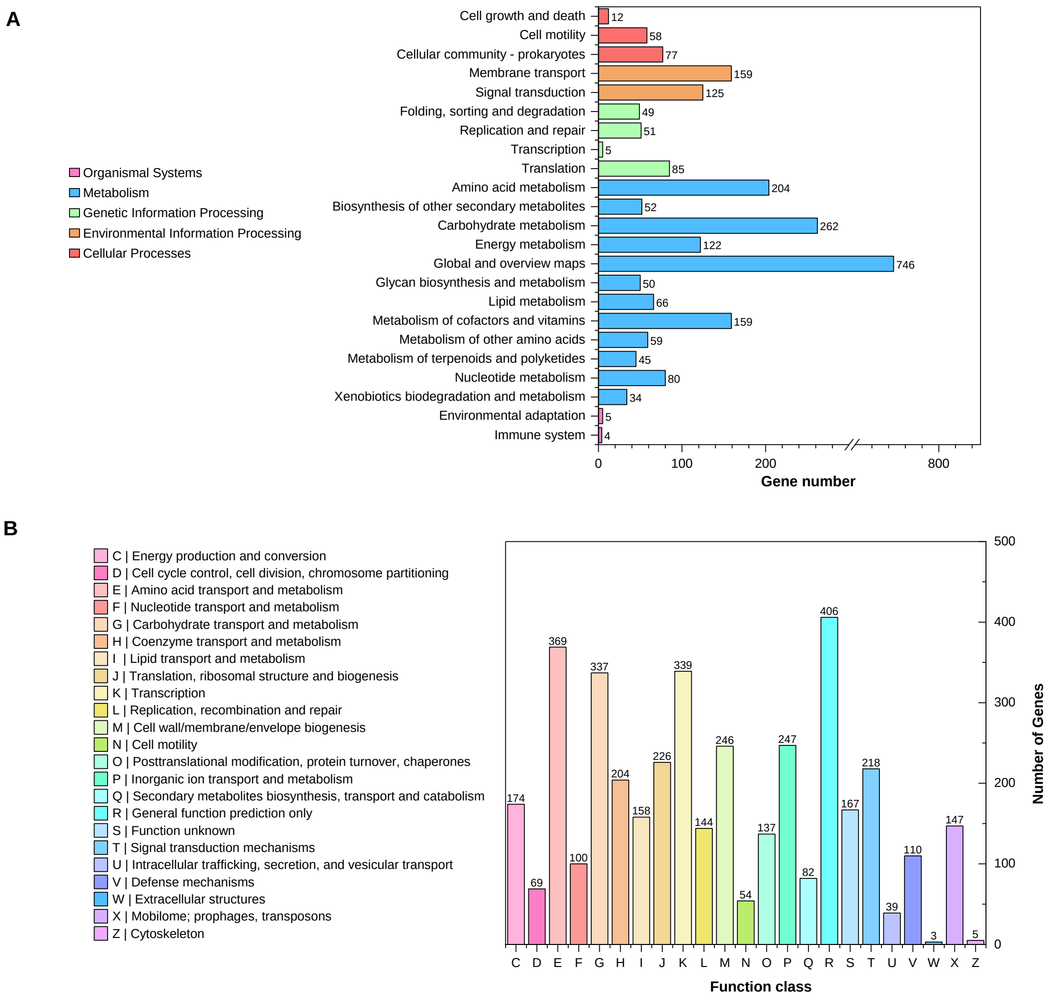

Fig. S1

A: The KEGG（Kyoto Encyclopedia of Genes and Genomes）functional annotation of *B. subtilis* AC-6

B: COG (Cluster of Orthologous Groups of proteins) functional annotation of *B. subtilis* AC-6.

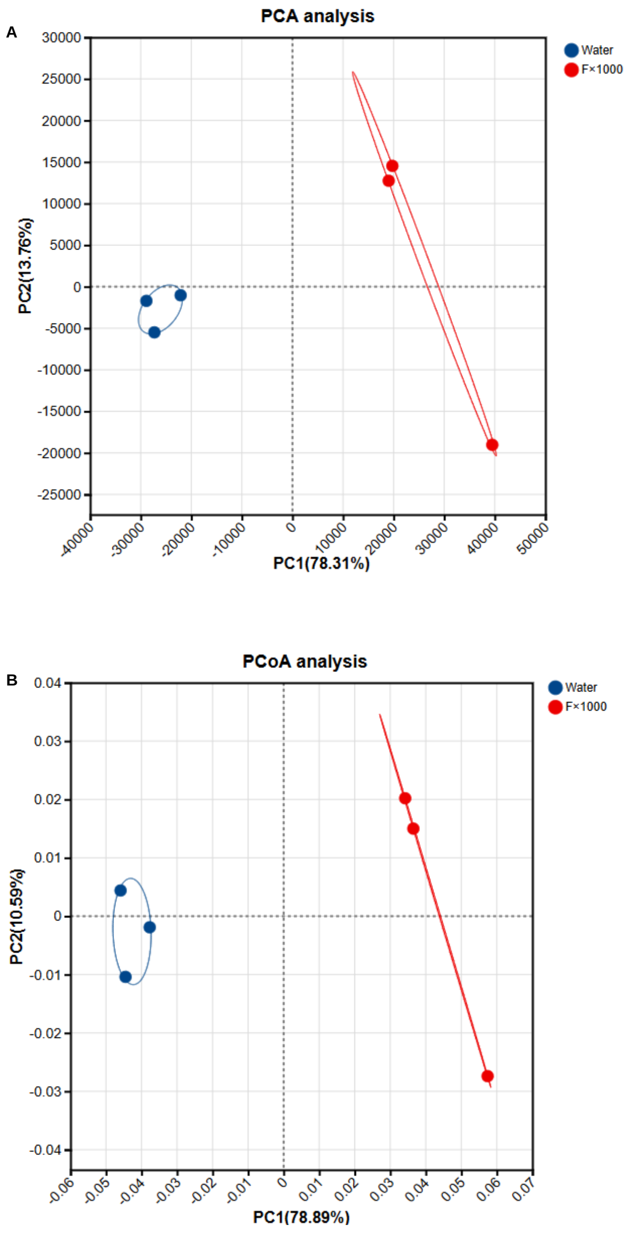

Fig. S2

Analysis of the rhizosphere soil microbial community of *B. rapa*.

A: Principal Component Analysis (PCA); B: Principal Coordinates Analysis (PCoA). Water indicates the control; F×1000 indicates the treatment with acetoin and 2,3-butanediol fermentation mixture diluted 1000 times.

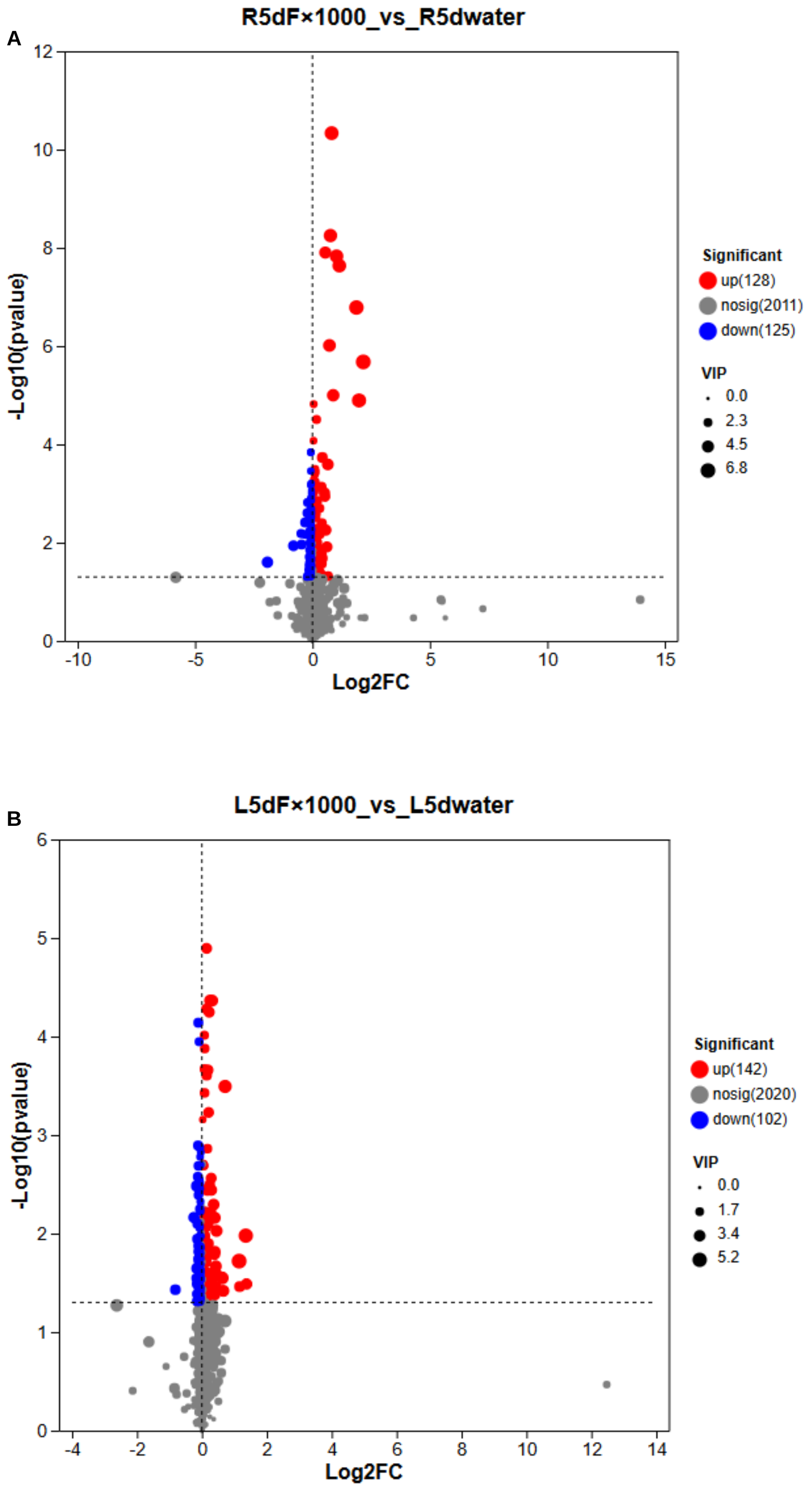

Fig. S3

Comparison of metabolites in *S. lycopersicum* var. under different treatments

A: Metabolite analysis in roots of *S. lycopersicum* var.; B: Metabolite analysis in leaves of *S. lycopersicum* var..

Water represents the control; F×1000 represents the treatment with acetoin and 2,3-butanediol fermentation mixture diluted 1000 times.

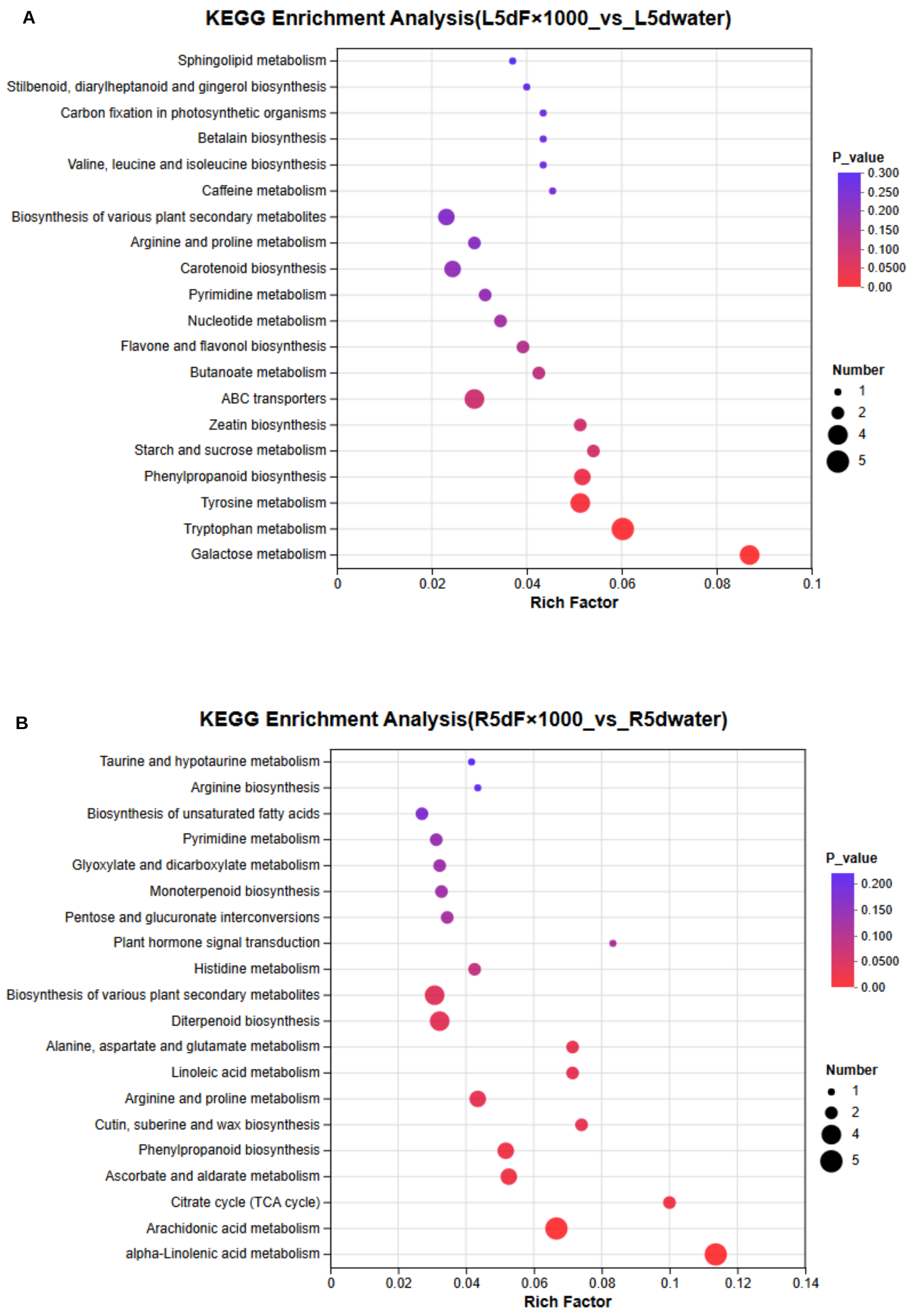

Fig. S4

Effects of the acetoin and 2,3-butanediol complex on metabolic pathway enrichment in *S. lycopersicum* var..

The y-axis represents KEGG metabolic pathways in *S. lycopersicum* var., and the x-axis represents the enrichment ratio. Water refers to the control; F×1000

refers to the treatment with the acetoin and 2,3-butanediol fermentation mixture diluted 1000 times. The size of each bubble indicates the number of enriched compounds mapped to the corresponding pathway, and the bubble color indicates the *p*-value significance level of pathway enrichment.
